# Computer-Aided Diagnosis Systems for Lung Cancer: Challenges and Methodologies

**DOI:** 10.1155/2013/942353

**Published:** 2013-01-29

**Authors:** Ayman El-Baz, Garth M. Beache, Georgy Gimel'farb, Kenji Suzuki, Kazunori Okada, Ahmed Elnakib, Ahmed Soliman, Behnoush Abdollahi

**Affiliations:** ^1^BioImaging Laboratory, Department of Bioengineering, University of Louisville, Louisville, KY 40292, USA; ^2^Department of Radiology, School of Medicine, University of Louisville, Louisville, KY 40202, USA; ^3^Department of Computer Science, The University of Auckland, Auckland 1142, New Zealand; ^4^Department of Radiology, The University of Chicago, 5841 South Maryland Avenue, Chicago, IL 60637, USA; ^5^Department of Computer Science, San Francisco State University, 911 Thornton Hall, 1600 Holloway Avenue, San Francisco, CA 94132, USA

## Abstract

This paper overviews one of the most important, interesting, and challenging problems in oncology, the problem of lung cancer diagnosis. Developing an effective *computer-aided diagnosis* (CAD) system for lung cancer is of great clinical importance and can increase the patient's chance of survival. For this reason, CAD systems for lung cancer have been investigated in a huge number of research studies. A typical CAD system for lung cancer diagnosis is composed of four main processing steps: segmentation of the lung fields, detection of nodules inside the lung fields, segmentation of the detected nodules, and diagnosis of the nodules as benign or malignant. This paper overviews the current state-of-the-art techniques that have been developed to implement each of these CAD processing steps. For each technique, various aspects of technical issues, implemented methodologies, training and testing databases, and validation methods, as well as achieved performances, are described. In addition, the paper addresses several challenges that researchers face in each implementation step and outlines the strengths and drawbacks of the existing approaches for lung cancer CAD systems.

## 1. Introduction

 Lung cancer remains the leading cause of cancer-related deaths in the US. In 2012, there were approximately 229,447 new cases of lung cancer and 159,124 related deaths [1]. Early diagnosis can improve the effectiveness of treatment and increase the patient's chance of survival [2]. *Positron emission tomography* (PET),* computed tomography* (CT),* low-dose computed tomography* (LDCT), and* contrast-enhanced computed tomography *(CE-CT) are the most common noninvasive imaging modalities for detecting and diagnosing lung nodules. PET scans are used to discriminate between malignant and benign lung nodules. Early detection of the nodules can be based on CT and LDCT scans that allow for reconstructing the anatomy of and detecting the anatomic changes in the chest. The CE-CT allows for reconstructing the anatomy of the chest and assessing the detected nodule's characteristics.

A wealth of known publications have investigated the development of *computer-aided diagnosis* (CAD) systems for lung cancer from a host of different image modalities. The success of a particular CAD system can be measured in terms of accuracy of diagnosis, speed, and automation level. The goal of this paper is to overview different CAD systems for lung cancer proposed in literature.

**Figure 1:**
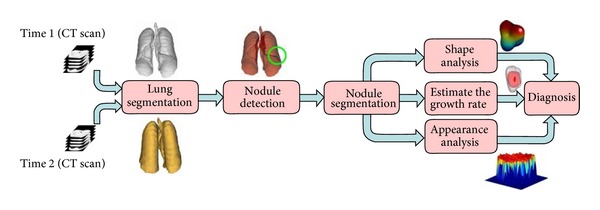
Typical *computer-aided diagnosis *(CAD) system for lung cancer. The input of a CAD system is the medical images obtained using an appropriate modality. A lung segmentation step is used to reduce the search space for lung nodules. Nodule detection is used to identify the locations of lung nodules. The detected nodules are segmented. Then, a candidate set of features, such as volume, shape, and/or appearance features, are extracted and used for diagnosis.

A schematic diagram of a typical CAD system for lung cancer is shown in Figure 1. The segmentation of lung tissues on chest images is a preprocessing step in developing the CAD system in order to reduce the search space for lung nodules. Next, detection and segmentation of lung nodules from the available search space are mandatory steps. Lastly, the classification of the detected nodules into benign and malignant is the final step. Classification of the detected nodules is a major component in CAD systems for detection and diagnosis of lung nodules in CT. In CAD systems for detection (often abbreviated as CADe), a classification component categorizes the nodule candidates identified in the previous step into nodules or nonnodules (i.e., normal anatomic structures), whereas a CAD system for diagnosis (often abbreviated as CADx) classifies detected nodules (either by a computer or a radiologist) into benign or malignant nodules.

Below, we will address each processing step in developing CAD systems: lung segmentation, nodule detection, nodule segmentation, and nodule diagnosis.

## 2. Lung Segmentation

 The segmentation of lungs from chest images is a crucial step in any CAD system that can lead to the early diagnosis of lung cancer, as well as other pulmonary diseases. The segmentation of lungs is a very challenging problem due to inhomogeneities in the lung region, pulmonary structures of similar densities such as arteries, veins, bronchi, and bronchioles, and different scanners and scanning protocols. A wealth of known publications has addressed the segmentation of lung regions from CT images and chest radiographs. The success of a particular technique can be measured in terms of accuracy, processing time, and automation level. Most existing techniques for lung segmentation can be classified into four categories: methods based on signal thresholding, deformable boundaries, shape models, or edges.

Healthy lung tissues form darker regions in CT images compared to other parts of the chest such as the heart and the liver. This fact has encouraged many researchers to search for an optimum threshold that separates the lungs from all other tissues. Hu et al. [3] computed iteratively such a threshold to get an initial lung region. Then the initial segmentation was refined by opening and closing morphological operations. This method was further used by Ukil and Reinhardt [4] and Van Rikxoort [5] to automatically segment the lung fields as a preprocessing step for lung lobe segmentation. Ross et al. [6] used a similar method to the Hu et al. approach [3] but used Otsu's method [7] for thresholding, instead of the iterative thresholding, in order to segment the lung fields as a step of lung lobe extraction. Yim et al. [8] extracted the lung fields by the region growing followed by connected-component analysis. Armato et al. [9, 10] used gray-level thresholding to segment the thorax from the background first and then the lungs from the thorax. A rolling ball filter was further applied to the segmented lung borders to avoid the loss of juxtapleural nodules. The identified lung fields were used to limit the search space for their lung nodule detection framework. In Pu et al. study [11], the threshold is selected automatically as described by Armato et al. [9]. A threshold-based region filling methodology was then used to segment the lung fields as a first step in a pulmonary fissure segmentation framework. Pu et al. [12] set a threshold to initially segment the lung regions. To refine the segmentation and include juxtapleural nodules, a border marching algorithm was used to march along the lung borders with an adaptive marching step in order to refine convex tracks.

Gao et al. [13] proposed another threshold-based segmentation approach consisting of four processing steps: (i) removing the large airway from the lung region by using isotropic diffusion to smooth edges followed by region growing, (ii) finding an optimal threshold to remove pulmonary vessels, (iii) separating the left and the right lungs by the detection of anterior and posterior junctions using the largest threshold, and (iv) morphological smoothing of the lung boundary along the mediastinum and lung wall based on the structure of the airway tree. To identify lung fields in a lung lobe segmentation framework, Wei et al. [14] selected a threshold to segment the lung regions using histogram analysis. The segmented lungs were then refined using *connect-component labeling* (CCL) and circular morphology closing. Ye et al. [15] used 3D adaptive fuzzy thresholding to segment the lung region from CT data. The segmentation was followed by smoothing the segmented lung contour, represented as chain code [16], by 1D Gaussian smoothing. They further applied a methodology to detect the lung nodules in the segmented lung fields.

The main problem of the threshold-based segmentation is that its accuracy is affected by many factors, including image acquisition protocol and scanner type (e.g., GE, and Siemens). Moreover, densities (in Hounsfield units) of some pulmonary structures, such as arteries, veins, bronchi, and bronchioles, are very close to densities of the chest tissues. As a result, the threshold-based segmentation cannot be accurate for the whole lung region and needs further intensive postprocessing steps to overcome the inhomogeneity of densities in the lung region.

Lung segmentation techniques of the second category use deformable boundary models, such as active contours (snakes), *level sets* (LS), or geodesic active contours. A snake starts from some initial position and shape and evolves under specific internal and external guiding forces to fit the shape of one or more desired objects. Snakes can extract a *region of interest* (ROI) or locate an object boundary. Itai et al. [17] extracted the lung region with a 2D parametric deformable model using the lung borders as an external force. The deformable model started from an initial segmentation obtained by a threshold estimated from CT data. The segmentation results were used as a preprocessing step to classify abnormal areas within each lung field. Silveira et al. [18] used a 2D geometric LS active contour being initialized at the boundary of the chest region, which was then automatically split into two regions representing the left and right lungs. The main drawbacks of the deformable model-based segmentation are the excessive sensitivity to initialization and the inability of traditional external forces (e.g., based on edges and gray levels) to capture natural inhomogeneity in the lung regions. As a result, it is hard to provide an adequate guidance to the deformable model to achieve the accurate segmentation.

To improve the segmentation accuracy, shape-based techniques add prior information about the lung shape to image signals. To use the shape prior, it should be aligned with the initial CT data before starting the segmentation. Annangi et al. [19] integrated a prior shape term, calculated as described in [20], with a term describing edge feature points and a term representing region-based data statistics [21] in a variational energy framework for lung segmentation. The formulated energy was used to guide an LS deformable model in order to segment the lung fields from *posterior-anterior* (PA) chest X-ray images. Shi et al. [22] used an adaptive shape prior to guiding a deformable model used to segment the lung fields from time-series data. The initial shape was trained from manually marked lung field contours from the population using the *principle component analysis* (PCA) method and was used to segment the initial time-point images of each subject. To address the shape variability for each subject, the shape was adapted for the segmentation of further time-point images with the previously segmented images from the same subject. Van Ginneken et al. [23] optimized the *active shape model* (ASM) developed by Tsai et al. [24] to segment the lung fields. They compared the segmentation with an *active appearance model*-(AAM-) based segmentation and a multiscale resolution pixel classification, concluding that the latter gave the best results. Hardie et al. [25] invoked the optimized ASM of van Ginneken et al. [23] to segment the lungs field in a CAD system developed to identify lung nodules on CT images.

Sun et al. [26] segmented the lungs in two main processing steps. First, a 3D ASM matching method is used to get a rough initial segmentation of the lung borders. Second, a global optimal surface finding method, developed by Li et al. [27], is used to find a refined smoothed segmentation of the lungs. Besbes and Paragios [28] used a graph-based shape model with image cues based on boosted features to segment the lung fields from chest radiographs. Sluimer et al. [29, 30] proposed to segment a pathological lung by using the shape model of a normal lung. Sofka et al. [31] aligned a shape model using a set of automatically detected anatomical landmarks and refined the shape model through an iterative surface deformation approach in order to segment lungs that involve pathologies. The main limitation of the shape-based segmentation techniques is that their accuracy depends strongly on how accurately the prior shape model is registered with respect to the CT image. Instead of using a shape prior, Kockelkorn et al. [32] used a user-interactive framework for lung segmentation in CT scans with severe abnormalities, where a user corrected the results obtained by a *k-nearest-neighbor* (KNN) classifier trained on prior data.

Hua et al. [33] presented an automatic method to segment pathological lung fields using a graph-based search of a cost function that incorporates the intensity, gradient, boundary smoothness, and the rib information. El-Baz et al. [34–36] proposed an iterative *Markov-Gibbs-random-field*-(MGRF-) based segmentation framework to segment the lung fields from LDCT images. A *linear combination of discrete Gaussian* (LCDG) model with positive and negative components [37, 38] was used to approximate the empirical distribution of the LDCT signals of the lung fields and their background, describing the first-order visual appearance model of the LDCT image. An initial segmentation of the lung fields was obtained by a voxel-wise Bayesian *maximum a posteriori* (MAP) classification of a given image, based on its LCDG approximation of the signals of the lung fields and their background. The segmentation of the lung fields was iteratively refined by the *iterative conditional mode* (ICM) relaxation that maximizes a MGRF energy that accounts for the first-order visual appearance model and the spatial interactions between the image voxels. They further extended their work by applying their iterative MGRF-based segmentation framework on different scale spaces [39, 40]. Then the segmentations of the different scales were fused using a Bayesian fusion approach to get the final segmentation of the lung region. Ali et al. [41] proposed a graph-cut segmentation algorithm for the lung fields based on the iterative MGRF-based segmentation in [34–36].

The edge-model-based lung segmentation is performed using spatial edge-detector filters or wavelet transforms. Campadelli et al. [42] detected an initial outline of lung borders by using the first derivative of Gaussian filters taken at four different orientations. Then, an edge tracking procedure using the *Laplacian of Gaussian* (LoG) operator at three different scales was used to find a continuous external lung contour, which was further integrated with the initial outline to produce the final lung segmentation from PA chest radiographs. Mendonca et al. [43] selected automatically the ROIs from PA chest radiographs as rectangular areas that surround each lung field as closely as possible through an iterative procedure. Edge points (i.e., the mediastinal, costal, top, and bottom edge points) were detected using spatial edge-detector filters and combined to define a closed contour for the lung borders. Korfiatis et al. [44] used 2D wavelet transform to highlight lung borders in a stack of 2D images. An optimal threshold, selected by the minimum error criterion [45], was applied to the wavelet-processed 3D stacks to segment lung volumes. 3D morphological processing was further performed to refine the final segmentation.

A review of the current methodologies for lung field segmentation is presented in Table 1. To efficiently reduce the search space for lung nodules, some technical issues should be further investigated to provide accurate segmentation of the lung fields. These technical issues include the automation level of the technique, the sensitivity of the method to the scanning parameters, the efficiency of an algorithm to work with different image modalities (e.g., CT, LDCT, or CE-CT), and the ability of the algorithm to provide a proper lung segmentation in cases with severe pathologies that are associated with inhomogeneities in the pathological lungs.

## 3. Detection of Lung Nodules

 After the definition of the search space for the nodules (e.g., the segmented lung fields), nodule detection is the next step in lung cancer CAD systems. Early detection of lung tumors (visible on chest radiographs as nodules) may increase the patients' chance of survival [1, 46], but the nodule detection problem is a complicated task; see, for example, [47, 48]. Nodules show up as relatively low-contrast white circular objects within the lung fields. The difficulty for CAD systems is to distinguish true nodules from (overlapping) shadows, vessels, and ribs.

At present, spiral LDCT is of prime interest for screening (asymptomatic, but high risk) groups for early detection of lung cancer [49–51]. The LDCT provides chest scans with very high spatial, temporal, and contrast resolution of anatomic structures and is able to gather a complete 3D volume of a human thorax in a single breath-hold [47]. Hence, for these reasons, in the recent years most lung cancer screening programs have been investigated in the United States [51–55] and Japan [48, 50, 56, 57] with LDCT as the screening modality of choice.

CAD systems for detection of lung nodules in thoracic CT (i.e., CADe) generally consist of two major stages: (1) initial candidate nodules are selected (i.e., identification of nodule candidates) and then (2) the *false positive nodules* (FPNs) are partially eliminated while preserving the *true positive nodules* (TPNs) (i.e., classification of the identified nodule candidates into nodules or nonnodules (i.e., normal anatomic structures)).

At the first stage, conformal nodule filtering [58] or unsharp masking [59] can enhance nodules and suppress other structures to separate the candidates from the background by simple thresholding (to improve the separation, the background trend is corrected in [60–63] within image regions of interest) or a multiple gray-level thresholding technique [9, 64, 65]. A series of 3D cylindrical and spherical filters are used to detect small lung nodules from *high-resolution CT* (HRCT) images [66–70]. Circular and semicircular nodule candidates can be detected by template matching [59, 71, 72]. However, these spherical, cylindrical, or circular assumptions are not adequate for describing general geometry of the lesions. This is because their shape can be irregular due to the spiculation or the attachments to the pleural surface (i.e., juxtapleural and peripheral) and vessels (i.e., vascularized) [73]. In [74–77], they used morphological operators to detect lung nodules. The drawbacks to these approaches are the difficulties in detecting lung wall nodules. Also, there are other pattern-recognition techniques used in the detection of lung nodules such as clustering [78–81], linear discriminate functions [82], rule-based classification [83], Hough's transform [84], connected-component analysis of thresholded CT slices [85, 86], gray-level distance transform [80], and patient-specific a priori model [87].

The FPNs are excluded at the second stage by nodule classification [60, 61, 84, 88–90]. The most popular way to do classification is to use a feature-based classifier. First, the nodule candidates identified in the first step are segmented, and features are extracted from the segmented nodule candidates. Features may include morphologic (or shape-based) features (e.g., size, circularity [61], curvature [90], etc.), gray-level-based features (including histogram-based features), and texture features. The task of the classifier is to determine “optimal” boundaries for separating classes (i.e., nodules or nonnodules) in the multidimensional feature space which is formed by the input features [91].

Feature-based classifiers include *linear discriminant analysis* (LDA) [92], rule-based or linear classifier [9, 63, 64, 66, 66, 68, 78, 81, 93], template matching [87], nearest cluster [75, 77], *Markov random field* (MRF) [94], *quadratic discriminant analysis* (QDA) [92], multilayer perceptron (often called just an *artificial neural network*—ANN) [74, 89, 95–97], and a *support vector machine* (SVM) [98, 99]. A classifier is trained with sets of input features and correct class labels. A class label of 1 is assigned to the corresponding output unit when a training sample belongs to that class, and 0 is assigned to the other output units. After training, the class of the unit with the maximum value is determined to be the corresponding class to which an unknown sample belongs.

Recently, as available computational power increased dramatically, *pixel/voxel-based machine learning* (PML) [100] emerged in medical image analysis which uses pixel/voxel values in images directly instead of features calculated from segmented regions as input information. Thus, feature calculation or segmentation is not required. Because the PML can avoid errors caused by inaccurate feature calculation and segmentation which often occur for subtle or complex lesions, the performance of the PML can potentially be higher for such lesions than that of common feature-based classifiers. PML includes neural filters [101, 102], convolution *neural networks* (NNs) [103–107] (including shift-invariant NNs [108–110]), and *massive-training ANNs* (MTANNs) [111–114] (including multiple MTANNs [111, 115–117], a mixture of expert MTANNs [118, 119], a *Laplacian eigenfunction MTANN* (LAP-MTANN) [120], and a *massive-training support vector regression* (MTSVR) [121]). Convolution NNs have been applied for classification tasks such as *false-positive* (FP) reduction in CADe systems for the detection of lung nodules in chest radiographs [103–105] and FP reduction in CADe systems for the detection of microcalcifications [106] and masses [107] in mammography. MTANNs have been used for classification, such as FP reduction in CADe systems for the detection of lung nodules in chest radiographs [115] and thoracic CT [111, 116, 122], distinction between benign and malignant lung nodules in thoracic CT [117], and FP reduction in a CADe system for polyp detection in CT colonography [112, 118–121].

Technical development of the classification step in CADe systems for the detection of lung nodules in CT is summarized in Table 2. In 1994, Giger et al. [123] developed a CADe system for the detection of lung nodules in CT. In their CADe system, classification was performed by geometric feature analysis in conjunction with a comparison of suspected regions in each slice with suspected regions in adjacent slices. In 1999, Armato et al. [9, 124] extended the method to include 3D feature analysis, a rule-based scheme, and LDA for classification. Gurcan et al. [78] employed a similar approach, that is, a rule-based scheme based on 2D and 3D features followed by LDA for classification. Lee et al. [71] employed a simpler approach which is a rule-based scheme based on 13 features for classification. Ko and Betke [64] differentiated between normal structures (vessels and bronchi) and nodules by the analysis of the candidates' location and shape using a rule-based classifier. Their method was able to detect nodules with a diameter larger than 3 mm and missed those with a diameter less than 3 mm or those that contacted the lung border.

Kanazawa et al. [81] segmented the nodule candidates (normal structures (vessels and bronchi) and nodules) within the lung fields using a fuzzy clustering method [209]. For each candidate, they extracted a shape, a gray-level, and a position features. Then, a rule-based filter was used to combine these features in order to detect the lung nodules. Brown et al. [87] build semantic network a priori models to describe the lung nodules and other structures. In the training phase, a set of features, composed of the X-ray attenuation range, the relative location, the volume, and a sphericity shape parameter, were used in the semantic network nodes to describe the expectation models for the lung nodules as well as other structures. For each test candidate, a fuzzy logic was used to score the match between the extracted candidate features and the priori estimated models to define its belonging to either nodule or other structures. Wiemker et al. [72] evaluated the compactness, thickness of connecting vessels, average Hounsfield (HU) value, and HU distribution within the nodule candidate to detect nodules using 1 mm HRCT slices. On 12 HRCT exams with 203 nodules, their method achieved a sensitivity of 0.86% and 4.4 FPs per case for nodules with a diameter ≥1 mm.

Mekada et al. [63] discriminated between nodule regions and normal structures (e.g., vessels) using the *maximum distance inside connected components* (MDCC) in 3D X-ray CT images. The number of FPNs was reduced by applying a minimum directional difference filter for the nodule candidates that have sizes smaller than a given threshold value. Their method achieved a sensitivity of 71% with an average number of 7.4 FP per case in a study composed of 242 CT medical exams. Awai et al. [74] identified the initial potential nodules using a sieve filter that selected the intrapulmonary structures larger than a predefined size as lung nodule candidates. Then, an ANN classifier was used to determine if the lesion is a true nodule or not based on a set of extracted candidate features, including the volume, roundness, average diameter, maximum diameter and the diameter perpendicular to it, and distance between potential nodule and thoracic. The sensitivity of this method was 80% and 0.87 FPs nodule per section on a test group composed of 82 CT exams (3556 sections) containing 78 nodules.

Paik et al. [69] used a method, called the *surface normal overlap* (SNO) method, to detect the lung nodules and colon polyps. The SNO method describes the shape and geometry of a potential nodule and assigns a score for each shape. A threshold score was used to discriminate between the lesions and other structures. Their method was tested on 8 lung CT datasets, achieving a varying sensitivity based on the allowed FPs per sets. At 1.3 FPs per dataset, a sensitivity of 80% was achieved; at 5.6 FPs per dataset, a sensitivity of 90% was achieved; and at 165 FPs per dataset, a sensitivity of 100% was achieved. Mendonca et al. [70] used a filter for highlighting the nodule-like structures (i.e., the ROI) in CT images. For every voxel in the ROI, the eigenvalues of a curvature tensor were computed and thresholds derived from anatomical models (i.e., a geometric and an intensity models) were used to label each voxel as spherical (e.g., nodules), cylindrical (e.g., vessels), or neither.

Suzuki et al. [111] developed an MTANN for the reduction of a single source of FPs and a multiple MTANN scheme for the reduction of multiple sources of FPs that had not been removed by LDA. This MTANN approach did not require a large number of training cases: the MTANN was able to be trained with 10 positive and 10 negative cases [210–212], whereas feature-based classifiers generally require 400–800 training cases [210–212]. Arimura et al. [116] employed a rule-based scheme followed by LDA or MTANN [111] for classification. Farag et al. [213, 214] and El-Baz et al. [125, 126, 215–218] developed a template modeling approach using LS for classification. Ge et al. [127] incorporated 3D gradient field descriptors and ellipsoid features in LDA for classification. Matsumoto et al. [128] employed LDA with 8 features for classification. Yuan et al. [129] tested a commercially available CADe system (ImageChecker CT, LN-1000, by R2 Technology, Sunnyvale, CA; Hologic now). Pu et al. [130] developed a scoring method based on the similarity distance of medial axis-like shapes obtained through a progressive clustering strategy combined with a marching cube algorithm from a sphere-based shape.

Retico et al. [131] used the MTANN approach (as they call it in their paper) for classification. Ye et al. [15] used a rule-based system followed by a weighted SVM for classification. Golosio et al. [132] used a fixed-topology ANN for classification and they evaluated their CADe system with a publicly available database from the *Lung Image Database Consortium* (LIDC) [219]. Murphy et al. [133] used a KNN classifier for classification. Tan et al. [135] developed a feature-selective classifier based on a genetic algorithm and ANNs for classification. Messay et al. [134] developed a sequential forward selection process for selecting the optimum features for LDA and quadratic discriminant analysis. Riccardi et al. [136] used a heuristic approach based on geometric features followed by an SVM for classification. Thus, various approaches have been proposed for the classification component in CADe systems.

 The above overview shows that some important factors should be further investigated in designing any CADe system for detecting lung nodules including the automation level, the speed, the ability of the detection scheme to detect nodules of different shapes, for example, irregular-shape nodules and not only the spherical ones, and the ability of the CADe system to detect cavity nodules, nodules contacted to the lung borders, and small nodules (e.g., less than 3 mm).

## 4. Lung Nodule Segmentation

Lung nodule segmentation refers to a task of delineating the spatial extent of focal nodular lesions appearing in chest CT scans, providing a critical foundation of CAD for lung cancers [220–222]. The nodule segmentation is a very important and crucial step in many lung cancer applications. In this section, we outline the clinical applications of lung nodule segmentation. Then, we review the state-of-the-art segmentation techniques for lung nodules from CT images as well as from PET images. Finally, we address various aspects of challenges that researchers often face in the development of techniques for solving the nodule segmentation problem.

### 4.1. Clinical Applications

 Accurate nodule segmentation is a crucial prerequisite for various diagnostic and treatment procedures for lung cancer [223], such as diagnosing tumor growth in follow-up CTs [140, 146], monitoring tumor response to therapy [224, 225], computer-aided lung cancer screening for early detection [71, 87, 226], and computer-aided diagnosis of tumor malignancy [115, 227]. In this application context, segmentation accuracy directly influences important clinical factors, such as the minimum size of measurable lesions and the shortest time duration for repeat CT in follow-up studies. Another interesting approach is to derive the standard RECIST/WHO 2D measures of lesions from the results of their volumetric 3D segmentation in order to improve their accuracy and reproducibility [177, 228].

The segmentation also defines a local image area from which image features can be extracted for further computational analyses. For example, lung cancer screening by CADe [71, 87, 226] often enhances the overall detection accuracy by segmenting detected nodules as a postanalysis to remove false-positive cases [229].

Malignancy classification of lung nodules in CADx [227] will also rely on accurate segmentation for extracting image appearance features whose quality dictates the overall classification performance [248]. Thus, improving the accuracy of nodule segmentation has a direct impact to these clinical tasks. While segmentation of a large solitary nodule can be straightforward, there exist types of nodules, such as small or partially solid nodules, which pose difficulty in accurate segmentation. Because these difficult cases are also of clinical importance (e.g., early detection of lung cancer with small nodules [249]; a partially solid nodule with high likelihood of being malignant [250–252]), nodule segmentation plays a critical role in successfully administering these clinical tasks.

### 4.2. CT Segmentation Techniques

 Due to the increasing clinical significance described above, the number of papers reported in the literature for pulmonary nodule segmentation has been increasing rapidly. The advent of thin-slice multidetector HRCT technologies in early 2000s has shifted trends in nodule segmentation research from early-thresholding-based 2D methods to more sophisticated flexible 3D/volumetric segmentations. Previously reported methods for lung nodule segmentation are summarized in Tables 3, 4, and 5. Prior to the advent of CT in routine medical practices, automatic detection, segmentation, and analysis of nodules in 2D chest radiography were actively investigated [253, 254]. Segmentation algorithms proposed in this context were intrinsically 2D based. During the early phase of CT applications, images are often made with a large slice thickness. Some early methods in the literature [141, 142] have also adopted this 2D approach for this reason. The following section summarizes the advances in nodule segmentation focusing on the recent volumetric approaches.

Technical approaches previously reported for volumetric lung nodule segmentation can be roughly classified into the following eleven categories: (1) thresholding [140–144, 146, 154], (2) mathematical morphology [73, 76, 147, 152, 153, 158], (3) region growing [152, 153, 175–178], (4) deformable model [137, 138, 160, 161, 163, 168, 182, 255], (5) dynamic programming [145, 169, 180], (6) spherical/ellipsoidal model fitting [148, 149, 151, 256, 257], (7) probabilistic classification [97, 156, 157, 166, 167, 174, 181], (8) discriminative classification [162, 183], (9) mean shift [150, 151, 170], (10) graph-cuts [172, 173], and (11) watersheds [165].

*Thresholding (TH).* TH is one of the most ubiquitous and straightforward methods for solving general segmentation problems. It yields a binary (foreground/background) segmentation of the *volume of interest* (VOI) by labeling each voxel by testing whether its intensity value surpasses a specific threshold value or not [16]. This approach was adapted by early methods proposed by Zhao et al. [142, 143] and Yankelevitz et al. [140, 141, 144]. Automatic data-driven methods to determine threshold values have been proposed by using K-mean clustering [140, 141] and average gradient magnitudes and boundary compactness [142, 143]. 

*Mathematical Morphology (MM).* MM is another popular technique in the lung nodule segmentation especially for handling special cases attached to nontarget structures such as vessels (juxtavascular) and parenchymal wall or the diaphragm (juxtapleural). MM is a set theoretic technique for processing geometric structures in binary and gray-scale images [16]. It offers various *morphological operations* (MOs) with four basic operators (erosion, dilation, opening, and closing) with a task-specific structuring element. Commonly, a sequence of iterative MOs are used to remove the nontarget structures juxtaposed to the target nodule in an initial binary segmentation result. Kostis et al. [73, 147] and Kuhnigk et al. [152, 153] have proposed effective iterative approaches for binary morphological filtering with various combinations of these basic operators. Okada et al. [158] presented a data-driven method to determine the ellipsoidal structuring element from anisotropic Gaussian fitting. Gray-scale MOs have also been successfully applied to nodule segmentation. Fetita et al. [76] proposed an approach with a *selective marking and depth-constrained* (SMDC) connection cost for handling the juxtaposed cases.

*Region Growing (RG).* RG is another classical image segmentation method that has been successfully adapted to the lung nodule segmentation problem. It identifies a connected-component region that surrounds a seeded pixel by iteratively adding neighboring pixels which satisfies a logical predicate defining pixel intensity proximity [16]. RG has been popular among the recent methods as their base component to produce initial rough segmentation to be improved on further, replacing the simpler TH adopted by earlier methods in the same context [140–144]. In the MM-based approach by Kuhnigk et al. [152, 153], RG was adopted in this manner. There are more recent studies [175–178] that have extended this approach as the main component of their overall segmentation algorithms. Dehmeshki et al. [175] proposed an adaptive sphericity-oriented contrast-based RG on the fuzzy connectivity map computed from the results of local adaptive thresholding segmentation. Diciotti et al. [176] presented an RG method with a fusion-segregation criteria using geodesic distances. Finally, Kubota et al. [177, 178] proposed an RG on an Euclidean distance map that is adjusted to handle juxtaposed structure more effectively. 

*Deformable Model (DM).* DM represents a class of segmentation methods based on the iterative evolution of contour curves that models the boundary of a target object, such as classic energy-minimization-based *active contour* (AC) [258], edge-based geodesic AC [259], and region-based variational LS [21]. One of the earliest works on volumetric lung nodule segmentation reported in the literature was by Kawata et al. [137, 138] which adopted the geodesic AC approach by Caselles et al. [139]. El-Baz et al. [160, 255] and Farag et al. [161] have adopted the energy minimization approach by Kass et al. [258] with a prior appearance model by MRF and a current appearance model by a bimodal LCDG. Farag et al. [182] proposed a variational LS solution with adaptive prior probability term for nodule segmentation. Yoo et al. [168] adopted the multiphase LS framework by Vese and Chan [260] to present an asymmetric 3-phase LS segmentation method for partially solid nodules. These approaches are adopted to evolve a 3D surface boundary directly. In Way et al. [163], an approach to derive volumetric segmentation by 2D ACs was applied to successive axial slices with 3D gradient, 3D curvature, and mask energy terms in order to facilitate continuity along slice depths. 

*Dynamic Programming (DP). *DP here refers to a variational energy minimization approach for detecting optimal contours in images [261]. It guarantees to find noniteratively the energy's global minimum among all possible contours, assuring its optimality [261, 262]. This global optimality is an attractive property of this approach leading to better reproducibility. DP has been successfully applied to detection, tracking, and matching the boundary of general objects in 2D images [262]. Xu et al. [145] also applied this to 2D nodule boundary detection with lesion contour discontinuity detection by transforming an image from the Cartesian to the polar coordinate system. An inherent issue to this approach is that its generalization to higher dimensional space is not straightforward. Several methods to extend this 2D approach to 3D surface detection for volumetric nodule segmentation have been reported. In Wang et al. [180], a sequence of 2D DPs are applied to successive slices with constraints for lesion center and radius from neighboring slices along the third dimension. This is repeated to the three orthogonal directions and the results are then fused. Wang et al. [169] proposed a method to transform a 3D VOI to a 2D image by transforming the 3D spherical to the 2D polar coordinate system along the points on the unit sphere sampled in the order of a spiral from the north to the south pole. After this spiral scanning transformation, the standard 2D DP was applied to detect 3D lesion boundary.

*Spherical/Ellipsoidal Model Fitting.* This fitting exploits the proximity of CT lung nodule appearance to the standard Gaussian intensity model. Such an approximation model with isotropic Gaussian has been used in an early work for CADe of nodules [71]. For segmentation, both ellipsoidal (anisotropic Gaussian) and spherical (LoG) models have been exploited to approximately segment and estimate the size of nodule lesions. Okada et al. [148, 149, 151] proposed a robust estimation method for fitting the anisotropic Gaussian intensity model (RAGF: *robust anisotropic Gaussian fitting*) by posing the problem as the scale selection over an anisotropic scale-space [149]. At each scale, the Gaussian model is fit to a nodule image by using the MS algorithm [263]. Then the most stable scale that minimizes the Jensen-Shannon divergence [264] computed over the varying scales determines the final outcome. In Diciotti et al. [257], the nodule size was estimated by using the multi-scale LoG filtering [265]. The characteristic scale defined over the LoG scale-space was adopted as the lesion's size estimate and as an initialization of their RG-based segmentation method [176]. Jirapatnakul et al. [256] also studied this method as their nodule size measurement.

*Probabilistic Classification (PC).* PC is another popular approach where each voxel is probabilistically classified as a nodule or other structures. Probability distributions such as class-conditional likelihoods and prior distributions for each class must first be estimated from data. At each voxel, the classification decision is then casted as the standard estimation framework, such as MAP, *maximum likelihood* (ML), and *likelihood ratio test* (LRT) [91]. Zhang et al. [97, 156] proposed an MAP approach by using the MRF as the prior and *Gaussian mixture model* (GMM) as the class-conditional model estimated by offline training [97] or online for each image [156]. Okada et al. [157] proposed an approach based on LRT where foreground and background likelihoods were estimated online over a joint spatio-intensity domain from the results of the RAGF [151]. In Zhou et al. [166, 167], likelihood distributions were estimated by nonparametric *kernel density estimator* (KDE), then the Bhattacharya distance was used as their classification criterion. Browder et al. [174] also proposed an ML approach for three classes (solid nodule, non-solid nodule, and parenchymal tissue), where a Gaussian model is used to define each distribution. In Tao et al. [181], likelihoods are derived by GMMs over a subspace found by LDA of various intensity features, yielding probability maps. Final segmentation is given by thresholding the map with a shape prior.

*Discriminative Classification (DC).* DC casts the segmentation problem as a voxel-wise classification similar to PC; however, the classifiers are built by using generic supervised machine learning algorithms without explicitly estimating probability distributions [91]. There exists numerous methods for supervised discriminative classifiers in the machine learning literature. For nodule segmentation, only a few approaches from them have been adopted. Van Ginneken [162] proposed a soft-segmentation method where a function is learned to map various-input intensity-based features computed at a voxel to a probability of the voxel being a part of a nodule. The output probability values for the training set were collected from multiple ground-truth segmentations. The KNN regression method was used to establish this function. Zinoveva et al. [183] proposed a similar soft segmentation method by using a decision-tree classifier with a *classification and regression tree* (CART) algorithm [266].

*Mean Shift (MS).* MS is a segmentation approach based on an iterative feature space analysis [263]. The MS algorithm performs a clustering of feature data points by iteratively seeking from each data point a mode of nonparametric distributions estimated by KDE [263]. Unlike the standard gradient descent algorithm [91], MS is provably convergent without requiring to tune the learning parameter thus can be implemented efficiently. Several works have adopted MS for the purpose of lung nodule segmentation. Okada et al. [150] proposed a robust nodule segmentation method that applied MS in the 4D joint spatio-intensity domain to refine the RAGF results, characterizing a nodule by an anisotropic Gaussian. Nie et al. [170] proposed an MS-based 2D nodule segmentation method over a feature space that combines the convergence index to the 3D joint spatio-intensity domain. Finally, the RAGF method proposed by Okada et al. [148, 151] extended the MS algorithm to the Gaussian scale-space [265] and applied it to estimate the covariance for robustly fitting a Gaussian to data.

*Graph-Cuts (GCs) [267] and Watersheds (WSs) [16].* GCs and WSs are the other well-known techniques of standard image segmentation that have been adopted to the nodule segmentation problem. Zheng et al. [172, 173] applied GC to derive their initial 2D nodule segmentation in their coupled segmentation-registration method with B-spline nonrigid registration [268]. Goodman et al. [165] utilized WS in their volumetry study. Each nodule was first segmented by using WS semiautomatically followed by a model-based shape analysis performed to determine anatomical characteristics of various nodule types.

The above-described techniques have been adopted to a number of commercially available semiautomatic software packages and put into the clinical practice today. Many reproducibility studies for lung nodule volumetry have investigated performance of such software packages [202, 269–273]. De Hoop et al. [274] compared six packages (Advantage ALA, GE, v7.4.63; Extended Brilliance Workspace, Philips, EBW v3.0; Lungcare I, Siemens, Somaris 5 VB 10A-W; Lungcare II, Siemens, Somaris 5 VE31H; OncoTreat, MEVIS v1.6; Vitrea, Vital images v3.8.1) and found that substantial variations in segmentation performance exist among current lung nodule software packages.

### 4.3. PET Segmentation Techniques

*Positron emission tomography* (PET) with the glucose analog, *18F-2-fluoro-2-deoxy-D-glucose* (FDG), has been widely used in oncology applications such as lung cancer detection and nodule segmentation. Using CT alone, target volume delineation of lung cancer is prone to interobserver variability, with variations in the *gross tumor volume* (GTV) definition being as high as 700% in lung tissue [275]. However, incorporating PET enhances the result of tumor outlining, diagnostic evaluation of pulmonary nodules, and staging the mediastinum. The widely used quantifier in PET imaging is the *standardized uptake value* (SUV) that estimates the intensity of the lesion on PET. The SUV is calculated either pixel-wise or over an ROI for each image at time *t*, as the ratio of the tissue radioactivity concentration, *c*(*t*), and injected dose at the time of injection divided by body weight:

(1) $$\begin{array}{c} \text{SUV}=\frac{c\left(t\right)}{\text{injected dose}\;\left(t_{0}\right)/\text{body weight}}. \end{array}$$

To define the tumor region, the most straightforward technique is to apply a thresholding-based method. Automatic thresholding-based methods used the SUV parameter to estimate the optimal threshold that defines the tumor region. Paulino and Johnstone [276] used an SUV value of 2.5 to autocontour the derived GTV. Other fixed thresholding-based methods define the tumor region by an arbitrary threshold value such as 40%, 42%, or 50% of the maximum SUV [189–193, 277].

In addition to the fixed thresholding-based techniques, there are other adaptive thresholding-based approaches that incorporate tumor volume, background activity, and source-to-background ratios [278–283]. Nestle et al. [278] compared different GTVs obtained from different methods to look for the optimal threshold value. Four different GTVs are obtained using four different methods: (1) GTV_vis_ obtained by visual interpretation, (2) GTV_40_ obtained by applying a threshold of 40% of the SUV_max_, (3) GTV_2.5_ obtained by applying a threshold equal to SUV = 2.5, and (4) GTV_bg_ obtained by using phantom studies as the best fit obtained based on the tumor and background intensities. GTV_vis_, GTV_2.5_, and GTV_bg_ showed a strong correlation with the CT-derived GTV, whereas the GTV_40_ was shown to be unsuitable. Nestle et al. concluded that the variability of the differences was due to the inhomogeneity in the nodules appearance and the difference in their sizes.

The main limitations of thresholding-based techniques are that they are user- and system-dependent and do not consider some important factors in the tumor delineation such as target motion due to respiration and cardiac activity. In addition, a single threshold model lacks the incorporation of other factors such as tumor size and the nonuniform distribution of FDG activity [278]. In many cases, due to conditions such as necrosis and hypoxia in *non-small-cell lung cancer* (NSCLC), a single threshold model cannot be obtained since these conditions create a non-uniform uptake value. Experimental measurements of radioactive spheres in a phantom using thresholding-based methods show that the thresholding-based methods are unreliable in the clinical studies [195, 278, 284, 285].

To provide more reliable tumor delineation, statistical segmentation techniques cast the tumor segmentation within a statistical framework as an unsupervised classification problem. For a given dataset composed of a set of items, a statistical classification framework attempts to label each item with some level of certainty, like that in [286]. For example, *fuzzy locally adaptive Bayesian* (FLAB) [197] and 3-FLAB [199] are locally adaptive Bayesian segmentation approaches that are combined with a fuzzy measure. Each voxel is assigned to its appropriate class based on its value and the values of its neighbors and also the noise model's parameters. In 3-FLAB which is an improvement of FLAB, three hard classes and three fuzzy transitions are incorporated and the model is evaluated on heterogenous tumors as well as homogenous ones. Based on unsupervised estimation and noise modeling, the *fuzzy C-means clustering method* (FCM) [287] and the *fuzzy hidden Markov chain* (FHMC) [196] similarly attempt to find large groupings within the intensity distributions obtained from the PET image. The segmentation results of these fuzzy-based methods show better tumor delineation with respect to the thresholding-based methods. However, they usually require an estimation of the initial class and they consider only the PET modality in their implementations.

More complex segmentation methodologies have been proposed to solve the lung tumor delineation problem [196, 197, 288–295]. For example, Li et al. [294] used an adaptive region growing method that extracts the tumor boundaries using deformable models in PET images. Avazpour et al. [198] used a region growing approach that is employed on coregistered PET/CT for the exclusion of collapsed lung. Mohamed et al. [296] and Woods et al. [297] incorporate textural and structural features in their segmentation methods. To summarize the approaches presented for the segmentation of lung nodules from PET images, Table 6 briefly describes the number of the patients enrolled in each study and the type of the nodule delineation approach with respect to the methodology, the approach dimension, and the approach automation level.

As PET acquisition takes several minutes, it is influenced by the patient's breathing and motion. These respiratory movements and cardiac actions result in the target motion which creates significant image blur that affects the accuracy of GTV estimation. On the other hand, using CT only implies a large uncertainty in the result of target volume delineation, especially in NSCLC [298, 299]. Reported cases, in which the GTV delineated based on CT, include abnormalities that appear totally devoid of FDG activity and can safely be removed from the GTV. Thus, the combination of PET and CT information has been studied in order to improve the target volume definition especially in NSCL and cases with atelectasis. In this regard, the recent studies have shown that the integration of PET information in the treatment planning has significantly reduced the interobserver contouring variability [298, 299].

To combine PET and CT information, a fusion technique should be applied to integrate both PET and CT images. The fusion techniques can be classified into one of three categories: (1) visual fusion in which both imaging modalities are simply considered side by side, (2) software fusion, and (3) hardware fusion. Using visual fusion, Kiffer et al. [187] showed that by using PET information the outlined volume has changed in 26.7% of the cases. They conclude that the variability on the volume estimation is due to the detection of abnormal mediastinal nodes on PET which cannot be detected on CT. Steenbakkers et al. [298] and Fox et al. [299] used a software fusion method and analyzed the observer variation in two phases, one with CT only and another one with fused PET/CT. The two studies addressed the issue of inter-observer variation reduction using matched PET and CT and concluded that the PET/CT software fusion is superior to visual fusion. Nestle et al. [189] and Munley et al. [188] used software fusion techniques that reported a significant change in the target volume extraction when compared to CT-defined volume. Nestle et al. [189] has documented that in 6 out of 17 patients with dystelectasis or atelectasis, the size of the delineated target was reduced with a median change of 19.3%. Munley et al. [188] reported an increase in the GTV in 34% of the cases when compared to CT. Erdi et al. [191] performed a study on patients who received CT and PET scanning using the same device. GTV, PTV, and normal tissues were initially contoured on the CT and then CT and PET were registered in a treatment-planning system. There was an average increase of 19% in the PTV volume in 7 out of 11 patients and an average decrease of 18% in the PTV in the other four patients. Van Der Wel et al. [194] showed that the GTV decreased significantly when shifting from the CT only to the fused PET/CT in 21 patients, thus allowing dose escalation. Further studies on the rate of recurrence when PET is used showed that only 1 out of 44 patients developed the tumor recurrence [300].

Table 7 summarizes the published studies on the effect of PET on GTV as a complementary to CT. For each study, the number of patients, the PET/CT fusion method, and the increase and decrease in the GTV as a percentage of the total number of the study cases are reported. These studies reported that the PET/CT fusion has improved the GTV estimation and thus is preferable for the treatment optimization in NSCLC. However, some well-known technical issues such as the resolution of PET, the exact tumor edge definition, and the misregistration between PET and CT images still need further investigations.

### 4.4. Nodule Segmentation Challenges

 Several challenges and aspects have been facing lung nodule segmentation techniques, such as the ability of a technique to segment the challenging types of nodules, the automation level of the technique, and its robustness. In this section, we briefly address each of these challenges.

#### 4.4.1. Nodule Types

 CT values for parenchymal tissues differ significantly from those for soft tissues. Therefore, segmentation of solitary and large solid nodules is technically straightforward. Problems arise when targeting (1) small nodules, (2) nodules attached to vessels (juxtavascular cases), (3) nodules attached to parenchymal wall and diaphragm (juxtapleural cases), and (4) ground-glass opacity nodules. The following outlines the nature of each issue and the current approaches handling them.

*Small*-nodule segmentation plays an important role for the early detection of lung cancers [249]. The advent of thin-slice HRCT has introduced the capability for the visualization of small nodules with less than 5 mm in diameter which could not be made visible by previous-generation CT technologies. Accurate segmentation of such small nodules is needed to assess malignancy of the lesions by measuring their growth rate as will be discussed in Section 5.1. *Partial-volume effect* (PVE) is the main technical concern when handling small nodules. Due to spatial discretization used for the CT imaging, a single voxel may represent more than one tissue type, resulting in averaging of their intensity values. This causes PVE, image blur especially at lesion boundaries, making their segmentation difficult. PVE becomes more pronounced when handling smaller lesions because the percentage of errors over the lesion volume would increase in such a case. This makes accurate area/volume measurement for small nodules more challenging. A number of approaches have been proposed to handle PVE in small-nodule segmentation [73, 146, 151, 153]. Ko et al. [146] presented the *partial-volume method* (PVM) for estimating nodule volume based on the consistency of the average attenuation amounts. Their phantom study demonstrated that PVM yields higher accuracy in volumetry than various thresholding methods. Kuhnigk et al. [153] proposed *segmentation-based partial-volume analysis* (SPVA) that extended the PVM approach to incorporate segmentation of VOI into the nodule core, the parenchyma area, and partial-volume region. A histogram sampled from the partial volume region was used to estimate the nodule's volume near its boundary. Kostis et al. [73] proposed isotropic resampling of volumetric images to mitigate PVE and also presented an upper bound for the PVE error of a perfect circle. Finally, RAGF proposed by Okada et al. [151] yields an ellipsoidal approximation of lesion boundary. When segmenting a small nodule, and volume measure derived directly from a fitted ellipsoid may be more accurate than voxel-wise segmentation results due to PVE.

Lung nodules are frequently attached to other pulmonary structures such as airways, blood vessels, parenchymal walls, and the diaphragm. Because the CT values of nodules and these nontarget structures are often very similar, accurate delineations of the extent of nodules from these structures become a difficult technical challenge.

*Juxtavascular* nodules refer to nodules that are attached to blood vessels. There are many studies that have addressed a solution for handling such juxtavascular cases [73, 76, 97, 143, 145, 151, 153, 165–168, 174–177, 185, 186]. One common approach for this purpose is the morphological filtering [73, 76, 97, 143, 153, 185]. Because the portion of nodules that attaches to vessels/airways is typically small with respect to the total extent of the 3D nodule surface, basic MOs, such as erosion, dilation, and opening, are often effective for most juxtavascular cases [76, 143]. More complex morphological filtering based on iterative [73, 185] and successive [153] combinations of these basic operators, convex hull operations [153, 177], and 3D moment analysis [174] have also been adopted as a postsegmentation refinement method. Geometric/shape-constrained segmentation is another popular approach in this context [151, 165, 175, 176, 186]. This approach integrates shape-based prior information within the segmentation process in order to bias the results toward a spherical/nodular shape and suppress the elongated nontarget structures attached to the target. Gaussian model fitting [151], eigen analysis of the Hessian matrix [166, 167], sphericity-oriented region growing [175], geodesics distance constraints between the connected components [186], and a steepest-ascent test [177] are some examples of this type of geometric-constraint approaches.

*Juxtapleural* nodules refer to cases that are attached to the parenchymal wall or the diaphragm. A number of studies have addressed a solution for handling such juxtapleural cases [73, 76, 145, 151, 153, 155, 158, 175, 177, 182, 184, 185]. *Pleural surface removal* (PSR) is the most common approach [73, 76, 153, 155, 158, 184, 185]. PSR can be addressed either globally or locally. The global methods first segment the entire lung from a CT image then use the result as a negative mask to avoid the non-target wall regions to be included in the nodule segmentation results. Morphological filtering was the common approach similar to juxtavascular cases [76, 153, 155]. In order to accurately segment lung walls, juxtapleural nodules must be discounted. Local surface smoothing [155] and convex hull operation [153] have specifically been adopted for this purpose. The local PSR methods perform the same task of removing the pleural surface within a VOI [73, 158, 184, 185]. Morphological filtering is also a popular approach in this context [73, 158, 185]. A local patch of the pleural surface can be approximated as a 3D plane. Kostis et al. [73] used a morphological filtering with a disk-shape kernel and Reeves et al. [185] presented an iterative clipping plane adjustment, exploiting this planar assumption. Beyond the planar model, a polynomial surface can be fit to VOI to improve the accuracy [184]. Another approach is to robustly detect the center of juxtapleural nodules because many reported general-purpose methods fail to do this. Prior constraint mean shift [158, 159], robust nodule core detection by centricity transformation [177], and the maximum curvature point [182] are the examples of robust methods addressing this task.

*The ground-glass opacity* (GGO) nodule refers to a type of nodules with subsolid CT values that are significantly lower than those of typical solid nodules. Based on whether any solid components are present or not, they are categorized into two types: *nonsolid/pure* and *partially solid/mixed*. Segmentation of the GGO nodules poses a technical challenge because it is hard to delineate their subtle boundaries and to model their irregular appearances. In clinical practice, the increased image resolution by the recent CT technologies have made it possible to study these small GGO nodules that were previously undetectable. Their growth is often very slow [252]; however, such GGO nodules, especially the mixed ones, have been found to have a high chance of being malignant [250]. Recent clinical studies have confirmed that they represent the histologic spectrum of peripheral adenocarcinomas, including the premalignant *atypical adenomatous hyperplasia* (AAH) and the malignant *bronchioloalveolar carcinoma* (BAC) [251]. A small non-solid GGO representing AAH or BAC can slowly evolve into an invasive lung adenocarcinoma over the period of 10 years [252]. Due to the clinical interests and technical challenge, many attempts have recently been made to propose the segmentation solutions for this nodule subtype [97, 146, 150, 156, 157, 166–168, 174, 177–179, 181]. The most common approach among them was the voxel-wise probabilistic classification in order to handle the subtle and irregular lesion appearances [97, 156, 157, 166, 167, 174, 181]. In this approach, segmentation is performed by assigning each voxel with a nodule/background label according to its probabilistic decision rule derived from the training data. MAP segmentation with a MRF prior [97, 156], LRT segmentation in the joint spatio-intensity domain [157], classification by the Bhattacharya distance with a nonparametric KDE-based intensity likelihood [166, 167], 3-class ML segmentation [174], and classification by the Otsu thresholding [7] with class-conditional probability map derived by an iterative LDA and shape-prior mask [181] are examples of various classification and modeling methods explored in the literature. Other interesting approaches for GGO nodule segmentation include the RAGF approach [151], asymmetric 3-phase LS segmentation [168], robust region growing [178], and graph Laplacian-based opacity map estimation [179].

Overall, the authors of the above-cited studies have agreed that the juxtapleural and part-solid GGO nodules are the most difficult types of nodules to segment accurately. *Developing type-specific and general nodule segmentation that can handle these difficult cases remains an unresolved challenge.*

#### 4.4.2. Automation

 In a CADx system, lung nodule segmentation serves as a subcomponent of the overall system. Beyond the obvious accuracy requirement, the usability of the segmentation methods plays a significant role in assessing the effectiveness of the overall system. In this sense, to reduce labor burden of users is one of the critical goals of the segmentation methods since an accurate but a labor-intensive method that requires a large amount of precise manual user interactions would be less preferred. In this application context, previously proposed segmentation methods are classified into two types: *automatic* and *semiautomatic*.

The automatic approach takes a CT image as an input then simultaneously segments all nodules present in the image without their locations specified by users [76, 154, 166, 167, 181]. Early methods with gray-scale MM filtering by Fetita et al. [76] and with automatic locally adaptive thresholding by Mullally et al. [154] have addressed this simultaneous segmentation of all nodules in volume. More recently, the probabilistic approach, targeting GGO nodule segmentation, has been exploited to address a couple of automatic segmentation methods. Zhou et al. [166, 167] used the Bhattacharya distance-based classification with a GGO intensity distribution modeled by the non-parametric KDE. Tao et al. [181] employed a class-conditional probability map modeled by a GMM over a subspace of various intensity features, such as *gray-level cooccurrence matrix* (GLCM), *local binary pattern* (LBP), and 3D Harr wavelet, derived by an iterative LDA. In both methods, the automation is realized by deriving a probabilistic model of general nodule appearance.

On the other hand, the semi-automatic approach assumes that the location of target nodules is known. In this assumption, a segmentation method takes a VOI as an input and assumes that the VOI contains the entire extent of a single nodule. Many segmentation algorithms fall into this category since their iterative process requires initializations or seeds. The amount of seeds required ranges from a single user click with robust model fitting [151] and region growing [151, 175, 177, 178] to an entire 3D contour with deformable models [137, 138, 160, 161, 163, 182]. Note that these semi-automatic methods can also be automated by using them together with a CADe nodule detection system which automatically provides seeds to one of these semi-automatic segmentations.

Error correction is another important usability aspect of nodule segmentation systems. As described in the previous section, there are methods for refining/correcting segmentation results for specific types of nodules that are difficult to segment, exploiting the nature of the specific nodule types. In order to improve their usability, such error correction process can be automated with an automatic detection of nodule types or of segmentation failures. Such nodule type-specific automatic error correction has been presented for juxtavascular [186] and juxtapleural cases [158].

Semi-automatic error correction is of interest in its clinical context. Such an approach can provide users stricter control on the details of segmentation outcomes in order to better exploit the domain knowledge of expert observers during its clinical workflow. Some segmentation algorithms allow users to interactively constrain segmentation results by specifying certain voxels to be a part of the results. For example, the optimization process used in the DP algorithm can take an arbitrary number of voxels as its hard constraints such that they are fixed to be a part of the final lesion contour. Xu et al. [145] exploited this pixel-wise hard constraint in their semi-automatic segmentation refinement by letting users specify correct contour voxels with mouse clicks.

#### 4.4.3. Robustness

 The semi-automatic procedure used in many segmentation methods involves user-determined seed points to indicate a target nodule to be segmented as described in the previous section. Different observers, or a single observer studying the same scan more than once, may produce different seed points, causing the intra- and interobserver variances with different segmentation results of the same nodule. Reduction of such variance plays a key role to realize repeatable and reproducible volumetry [301]. For example, in percentage error of estimated volume, this inter-observer variance can be as high as 20% [225]. A number of robust approaches have been studied to design a reliable and robust segmentation solution against such variabilities [151, 153, 175, 177, 178, 184]. One of the common approaches in this context is to robustly estimate nodule's center/core from a user-specified seed in order to reduce the intra- and inter-observer variance of the semi-automatic methods. The result of this process can be treated as the *optimal seed* that refines the user-specified seed to be robust against the perturbations due to user interactions. RAGF proposed by Okada et al. [151] estimated a nodule center by majority-voting of convergence of the mean shift procedures initialized by voxels randomly sampled around the initial seed. Kuhnigk et al. [153] detected the optimal seed as the convergence of an iterative local maximum search of 3D distance map around the initial seed. Dehmeshki et al. [175] chose the optimal seed as the voxel of the highest intensity value among the maximum distance voxels in a 3D distance map. Finally, in Kubota et al. [177, 178], the optimal seed was estimated by the voxels with maximum centricity values computed over a 3D distance map.

Some common robust estimation techniques have also been adopted to the nodule segmentation problem. A model fitting process can be made robust by ignoring outlier samples and only considering inlier samples. This standard principle has been applied in the RAGF method by fitting an anisotropic Gaussian only with samples within a basin of attraction defined by the MS [151], and in the robust lung surface modeling by fitting a polynomial surface to the lung wall only with samples that lie on the pleural surface but not on the nodule [184]. A perturbation-based stability analysis was adopted in the RAGF method by Okada et al. [151] in order to determine the most stable scale for a Gaussian scale-space by minimizing the Jensen-Shannon divergence computed with a set of the Gaussians estimated with perturbed scales.

As discussed in Section 4.4.1, variability among various types of nodules poses a significant challenge for accurate nodule segmentation. Some robust approaches to handle these various nodule types have been proposed yielding general-purpose segmentation methods. The RAGF method [151] imposes the ellipsoidal constraints for handling juxtaposed cases. A similar approach with LoG filtering, imposing the spherical constraints of various sizes, has also been applied for the detection and rough segmentation of nodules [256, 257]. Recently, Kubota et al. [177, 178] have proposed a robust region growing method that successfully handles all types of nodules.

#### 4.4.4. Validation

Validation refers to the experimental procedures that measure the accuracy of segmentation methods to be evaluated. It is important not only for choosing the best performing method for a particular application but also deriving some critical clinical parameters, such as the minimum size of measurable lesions and the shortest time duration for repeat CT in follow-up studies [185, 225]. Designing the effective validation procedure is a major challenge in the lung nodule segmentation research. This is because manual lesion contouring by observers used to prepare the *ground-truth* (GT) segmentations is labor-intensive, making it difficult to create large GT datasets. Another reason is the intra- and inter-observer variability for nodule segmentation, indicating that even the manual GTs prepared by expert radiologists can vary significantly [301].

The most accurate gold standard for validation currently available is chest phantom scans, where an artificial device simulating nodules and other parenchymal structures of known sizes is imaged by a standard CT scanner. Many studies have utilized various phantom scans for tuning parameters and bench-marking their best possible performance in vivo [140, 141, 146, 153, 154, 164, 176, 204, 302–304]. Recently, El-Baz et al. [305–307] validated the growth rate volumetric measurements on elastic lung synthetic phantoms. They developed more realistic phantoms using a state-of-the-art microfluidics technology to mimic the contractions and expansions of the lung and nodules seen during normal breathing.

Experimental validation of nodule segmentation methods with actual patient scans poses difficulty as described above. One common approach is based on subjective judgment of the segmentation results by visual inspection of expert observers. In this approach, the observer(s) classifies each case as success or failure, then the rate of successfully segmentation is reported [73, 97, 150, 151, 153, 165, 175, 176, 182, 184, 186]. Some authors prepare and use GT datasets of 2D segmentation [142] and of 3D segmentation for a solid nodule [154, 160, 172, 177] and for GGO nodules [156, 174, 177, 179, 181]. With such GTs, various segmentation methods have been validated by a number of quantitative accuracy and error measures, such as (1)  *overlap ratio* (a fraction of cardinality of the intersection and the union of voxel sets for a lesion's segmentation and its GT) [156, 162, 163, 169, 170, 177, 180, 181, 183], (2) *percentage voxel error rate* (percentage of voxels missegmented with respect to the total number of voxels in a nodule) [160, 163, 172, 180], and (3) *percentage volume error rate* (percentage of error in volume measurement with respect to the GT's volume) [154, 162, 176]. The mean, standard deviation, and the root mean square statistics are often reported for these accuracy/error measures computed for a set of test cases.

Publicly available nodule CT datasets with GT segmentations are an important way to facilitate better validation, especially for cross-algorithm comparisons. In 2003, the *Early Lung Cancer Action Program* (ELCAP) [249] made a dataset of chest CT scans exhibiting lung nodules available to the public [308]. This dataset has only been used in a few recent studies [174, 182]. It was the LIDC that made the most significant efforts to make such nodule CT datasets with GTs publicly available [219, 309]. Currently two datasets covering many types of nodules with multiple GT segmentations for each case are available through their website [310], which have already been used by many studies since 2005 [162, 163, 169, 176, 177, 180, 183, 186]. More recently, Italung dataset from a lung cancer screening program in Italy [311, 312] has been used in the studies by Diciotti et al. [176, 186]. Despite the increased availability of these public datasets, comprehensive comparisons of various methods proposed previously have not yet been thoroughly investigated. Kubota et al. [177] have reported a comparison of five leading methods with the LIDC datasets in their recent report; *however, more comprehensive comparative studies are necessary for establishing the state-of-the-art in this active research field*.

## 5. Diagnosis of Lung Nodules

Once the lung nodules are detected and segmented from the corresponding chest images, the next task is to determine whether the detected nodules are malignant or benign. A number of researchers developed CADx systems for this task, which distinguish malignant nodules from benign nodules automatically and/or determine the likelihood of malignancy for the detected nodules based on the geometrical size, shape, and appearance of the nodules. The performance of systems was generally evaluated by using the *receiver-operating-characteristic* (ROC) analysis [313], because this task is a two-class classification. The area under the ROC curve (*A*_*z*_) [314] was often used as a performance index. Since the malignancy of lung nodules correlates highly with their geometrical size, shape, and appearance descriptors, we will review the diagnostic systems that are based on each of these descriptors.

### 5.1. Diagnosis of Lung Nodules Based on Growth Rate

 Growth of small pulmonary nodules measured in 2D area [141] and 3D volume [140] has been shown to predict the malignancy in early clinical studies [140, 141, 315–317] and monitoring the tumor response to therapy [224]. A more recent clinical lung cancer screening study [318] has demonstrated the effectiveness of doubling time measured with commercial nodule segmentation software in diagnosing adenocarcinoma and bronchioloalveolar carcinoma. In oncology, there exists standard clinical protocols for measuring tumor size, such as RECIST and WHO [224]. However, these measures are based on crude linear (sum of 2D maximal diameters) or bilinear (product of 2D maximal diameter and the largest perpendicular length) approximation of 2D nodule areas, developed prior to the advent of the HRCT technologies. Approximation errors of these 2D measures limit the minimum size of measurable lesions (e.g., 10 mm in RECIST) because of the well-known *volume averaging* effect in which growth of smaller nodules cannot be determined with accuracy by them [224]. Addressing this shortcoming, segmentation-based volumetric measures have recently been reported in the clinical literature and actively investigated as alternative to these traditional 2D measures for more accurate size measurement. Accurate volumetric measures as results enable lesion's *volumetry*: objective quantification of lesion's growth in terms of growth rate (i.e., differentiating nodule's volume measured at different time-points) and/or doubling time (i.e., time that it takes to double the volume of a lesion) [185, 225].

The aforementioned RECIST and WHO measures involve observer's subjective judgment of 2D lesion boundary and manual measurement of lesion's diameters with a ruler or electronic calipers by oncologists/radiologists [224]. Exercising the same approach to the volumetry would necessitate manual contouring of lesion boundary by the trained observers, which significantly increases their labor burden. Furthermore, the subjective nature of such manual measurements inevitably causes significant intra- and interobserver variance. In a study comparing various lesion size metrics derived from manually segmented lesion boundaries [301], all 1D, 2D, and 3D/volumetric metrics, included in this study, resulted in high inter-observer variations, mitigating reproducibility of the measures. Reduction of such intra- and inter-observer variance and observer's burden are major motivations for adapting automatic methods for lesion segmentation in order to facilitate objectivity in the volumetry [165, 185].

A large volume of the recent studies have addressed reproducibility/repeatability of lung nodule volumetry by characterizing its bias and variance with respect to a number of variational factors relevant to current clinical practice and workflows. Factors considered in these studies include nodule characteristics such as size [147, 204, 269, 303, 329, 330], shape [269, 330], and appearance types of solidness [273]; pulmonary deformation due to cardiovascular motion [331] and inspiration [269]; CT reconstruction parameters such as slice thickness [164, 204, 304, 329, 330, 332], slice intervals [164, 204, 304, 332], field of views [164, 204, 304, 332], algorithm types [146, 164, 332], kernel types [273, 304], tube current time [146, 164], and dosage settings [271]; CT scanner vendors [303]; segmentation performance due to choices of threshold parameters in a segmentation algorithm [204, 248], segmentation algorithms [146, 248, 302, 333], segmentation algorithms in the same segmentation software package [272], segmentation software packages [274], and versions of a segmentation software package [270]; intra- and interobserver variations by using a commercial software package for semi-automatic nodule segmentation and volumetry [165, 202, 269]; levels of observer's experience and training [302].

Results of these studies have revealed considerable variability of the current volumetric measures when the above-listed factors are altered during the volumetric measurement process over time. These variations directly limit the shortest time interval of CT follow-up studies to be some large values, reducing its clinical usability [147]. Note that a fixed-value bias in segmentation error is canceled out when measuring volumetry so that even an inaccurate segmentation algorithm can be a good choice for volumetry as long as it is reproducible and robust [154]. Thus a robust segmentation algorithm that produces more consistent results than the existing accurate but less robust solutions can be a better choice in this application context [151, 224].

*Technical Approaches*. Volume measurement precision and accuracy depend on a number of factors, including the interscan variability, slice selection artifacts, differences in degree of inspiration and scan angles, and performance of nodule segmentation algorithms, which can make comparing serial scans unreliable. Below, we provide an overview of the existing work on measuring the growth rate of the detected lung nodules.

Generally, the growth rate of pulmonary nodules is determined by a size-based comparison of different temporal CT scans. Earlier 2D techniques exploited changes in the maximal transverse diameter of the nodule to estimate the growth rate between the CT scans [123, 141, 142, 231, 334]. Unfortunately, these techniques met with problems; for example, possible asymmetric growth results not only in minimal changes in the maximal diameter, but also in an increase of the overall lesion volume [201]. Hence, alternative 3D approaches were published for the measurement of the growth rate of small nodules. These volumetric measurements [140, 146, 147, 172, 185, 200, 202–207, 225, 335–338] have overcome the former 2D limitations.

Yankelevitz et al. [140] used HRCT scans to assess the growth rate of small pulmonary nodules. The ROI containing the nodule was identified in each image manually by a radiologist. Then it was resampled to obtain an isotropic space using a 3D linear interpolation, thresholded, and segmented using a 3D technique to reconstruct the 3D image volume. The number of voxels contained in the resulting volume was counted, and the doubling times were estimated using a simple exponential growth model. This 3D method offered an advantage over the 2D counterpart that measured the cross-sectional area, but it did not take into account the global motions of the patients due to their movements and the local motions of the whole lung tissues due to breathing and heart beating.

Reeves et al. [185] presented a method for measuring the change in the nodule size from two CT images obtained close in time where the ROI of each CT scan was selected by hand and resampled to an isotropic space. To make an accurate assessment and facilitate the comparison of the selected regions, a registration process using the 3D rigid-body transformation was performed such that both nodules would have the same position and orientation in the image space. Following the registration stage, an adaptive thresholding technique for segmenting the nodule was applied. A rule-based segmentation adjustment was applied to both nodule segmentations. By comparing the nodule segmentations and the thresholded regions, this rule-based system achieved a more consistent measurement of the nodule volumes by discarding missegmented nodule voxels. The main limitation of this work is that only the global motion of the patient, but not the local motion due to breathing and heart beating, was taken into account. This strongly affects the estimated growth rate, especially for small detected nodules (less than 5 mm in diameter).

Taking into account the difference in inspiration levels, Zhao et al. [142] presented an *adaptive doubling time* (ADT) measure of the growth rate of the detected lung nodules. The ADT calculation was obtained through non-rigid lung registration that took into account expanding or shrinking the nodule. This was accomplished by weighting matching costs of each voxel based on a proposed nodule detection process and a segmentation refinement process. The main limitation of this framework is that the nonrigid registration is applied directly to the segmented nodules. This affects the growth rate estimation because after segmentation of the lung nodules, we can no longer discriminate between the changes due to the true growth rate of the lung nodules and the changes in their shapes that come from breathing and heart beating.

Kawata et al. [336] coregistered the pulmonary nodules by using rigid-body registration and affine registration at two different stages. The nodules were segmented using a 3D deformable surface model, and curvature features were calculated to track the temporal evolution of the nodule. The same research group presented an extension of [336] by adding a 3D non-rigid deformable registration stage, and the analysis was performed using a displacement field to quantify areas of the nodule growth over time [337]. Zheng et al. [172] proposed a simultaneous segmentation and registration of the lung to measure the growth rate from serial CT data. They used a non-rigid transformation for lung deformation and rigid structure for the tumor in order to preserve the volume and the shape of the tumor during the registration. Segmentation of the 3D lung and tumor was based on a 2D graph-cut algorithm, and a B-spline-based non-rigid registration was used. Both of these works have the same limitation as the above-mentioned work of Zhao et al. [142].

Jirapatnakul et al. [206] presented a nodule growth measurement method, called *growth analysis from density* (GAD). They applied a Gaussian weighting function to the region around the nodule to reduce the influence of structures lying far from the nodule center. Also, some researchers used a number of commercial packages that have been released by the CT vendors for measuring the volume of pulmonary nodules, and a number of studies have evaluated the accuracy and limitations of these software packages. Since the actual volumes of real pulmonary nodules are unknown, such evaluations usually involve either radiologists/experts as the “gold standard” [200, 202, 203, 205, 207] or synthetic phantoms for which the volumes of the nodules are known [146, 204, 305–307, 338]. A general limitation of the majority of the volumetric measurement algorithms is that they are only capable of segmenting solid nodules. Moreover, the results from these packages show that the volumetric error depends on the performance of the segmentation algorithms, particularly in the presence of the nodule's vascular and pleural attachments [225].

Recently, El-Baz et al. [208, 339–345] proposed a method for monitoring the development of lung nodules detected in successive chest LDCT scans of a patient. To accurately monitor the volumetric changes between the corresponding nodules, a two-step registration approach was applied [346]. First, a global alignment of successive LDCT scans was performed using the learned LDCT prior appearance model in order to maximize the overlap between the scans. Second, a local registration step was performed to handle the local motion caused by breathing and heart beating. This step is based on deforming the target object over evolved closed equispaced surfaces to match a prototype. Preliminary results on the 135 LDCT datasets from 27 patients showed that their two-step registration methodology could lead to accurate growth rate measurements and thus more precise diagnosis of the lung nodules.

Table 8 briefly overviews the different growth rate techniques. In summary, several aspects of growth rate techniques should have further investigations. One of these aspects is to consider the global motion of the patients due to their movements and the local motions of the whole lung tissues due to breathing and heart beating in the volumetric measurements of growth rate. Another aspect is that the application of global and local registration directly to the segmented nodule leads to the inability to discriminate between the changes due to the true growth of the lung nodules and the changes in the nodule shape which come from breathing and heart beating. Finally, special types of lung nodules such as cavities and ground-glass nodules cannot be diagnosed using the current growth-rate techniques, so further methods and nodule descriptors are needed.

### 5.2. Diagnosis of Lung Nodules Based on Shape and Appearance

A great deal of work has been published regarding the usefulness of morphologic features to distinguish between malignant and benign pulmonary nodules on CT and, to a lesser extent, chest radiographs. Several studies have shown a correlation between different nodule shape characteristics and their underlying pathology. For example, Furuya et al. [347] analyzed the margin characteristics of 193 pulmonary nodules on HRCT scans and subjectively classified them as one of several types, including round, lobulated, densely spiculated, ragged, and halo. They found a high level of malignancy among the lobulated (82%), spiculated (97%), ragged (93%), and halo (100%) nodules, while 66% of the round nodules proved to be benign.

Automatically extracted features have also been shown to correlate with underlying malignancy. Kawata et al. [137, 227] quantified the surface curvature and the degree of surrounding radiating patterns in biopsy-proven benign and malignant nodules when compared with the resulting feature maps. Their results showed good separation of the feature maps between the two categories. Their further work [348] extended the curvature analysis method to include internal nodule features, and using this method, which is described in more detail below, they attained similar results. The same research group [233] designed an automated retrieval system to obtain diagnosis and prognosis information by searching similar images in a 3D CT image database of pulmonary nodules (248 nodule, 179 malignant and 69 benign) for which the diagnosis is known. An ROI is selected to include the nodule region and its surrounding. Each voxel in the ROI is represented using its CT density and a curvature shape index. The CT density and the shape index are characterized using joint histograms for analysis. For each input nodule, a similarity measure between the input nodule and the database is estimated using the correlation coefficient of the joint histograms of the nodules. The results for querying the 3D database for similar nodules show a reasonable set of similar nodules sorted from highest to lowest similarity with the queried nodule. Similarly, fractal analysis has been used to quantify the nodule margin characteristics of benign and malignant nodules. Kido et al. [349] used 2D and 3D fractal dimensions to analyze the lung-nodule interface in a series of 117 peripheral pulmonary nodules with various underlying pathology, including benign hamartomas, tuberculomas, and pneumonias, as well as malignant diagnoses including bronchogenic carcinomas. They noted statistically significant differences between the 2D fractal dimensions of hamartomas and all other nodules, as well as differences between the 3D fractal dimensions of pneumonias and tuberculomas and bronchogenic carcinomas. Although none of these studies directly assessed the accuracy of their methods for diagnosis prediction, they supported the notion that the nodule shape can potentially be used by automated systems to distinguish between benign and malignant nodules.

Several groups have designed CAD systems with the goal of predicting a diagnosis based on features extracted from CT scans or chest radiographs. In general, these systems share the following common schema: first extracting features from the images, then designing and using an automatic classifier to categorize nodules based on these features, and lastly, evaluating the performance of the system with ROC analysis. The CAD systems differ in the specific extracted features and the type of classifier used, with *linear discriminant classifiers* (LDC) and *neural networks* (NNs) being the most common. Below, systems based on LDC classifiers will be discussed followed by systems based on NNs and other types of classifiers.

Kawata and colleagues [232] designed a CT-based CAD system that classified pulmonary nodules based on a combination of the curvature index and the relationship of the nodules to their surrounding features. The curvature index of a nodule is calculated from a combination of shape indices, which describe the surface type (i.e., ridge, saddle, pit, etc.), and curvedness, which describes the degree of curvature. The area surrounding the nodules was assessed for the degree of vascular convergence and pleural retraction using vector field analysis. Using an LDC classifier based on these features to evaluate a series of 248 nodules (179 malignant and 69 benign), they found the combination of curvature-based and surrounding features to be most accurate (area under ROC curve (*A*_*z*_ = 0.94)), followed by curvature-based alone (*A*_*z*_ = 0.88), and surrounding characteristics alone (*A*_*z*_ = 0.69). Mori et al. [241] also designed a CAD system using curvedness index in combination with dynamic contrast-enhanced CT in order to evaluate the temporal change as a possible discriminating feature of benign and malignant nodules. Shape index, curvedness values, and attenuation were calculated at 0, 2, and 4 minutes after contrast administration, and using these values, a score was generated by an LDC. Attenuation had an *A*_*z*_ value of 0.69 at 2 minutes after contrast, the highest of the three time points. Curvedness yielded a maximum *A*_*z*_ of 0.83 at 2 minutes, and the shape index had an *A*_*z*_ value of 0.90 at 0 and 2 minutes. The combination of all three features had an *A*_*z*_ value of 1.00 at 4 minutes.

The CAD system developed by McNitt-Gray et al. [231] used a pattern classification approach to determine the malignancy of pulmonary nodules on HRCT in a series of 31 cases (17 malignant, 14 benign). They identified solitary nodules using a semi-automated contouring technique and extracted quantitative measures of the resulting contour related to shape, size, attenuation, distribution of attenuation and texture. Using a stepwise discriminant analysis, they selected features that were best able to predict malignancy and used these to design a LDC to characterize the nodules. The selected features predicted malignancy with an accuracy of 90.3% (28/31); however, no *A*_*z*_ value was reported.

Shah et al. [350] designed a CAD system that extracted features from two separate contours, one including only the solid portion of the nodule and one including any ground-glass components. For each contour, 75 features were calculated to measure nodule attenuation, shape, and texture. These features were then inputed into a feature selection step, and four different classifiers were used to determine if the diagnosis could be predicted from the feature vector. Training and testing was conducted using both resubstitution and leave-one-out methods. With leave-one-out testing methodology with a database composed of 19 malignant and 16 benign nodules, the classifiers resulted with an *A*_*z*_ ranging from 0.68 to 0.92. When evaluating with resubstitution, the *A*_*z*_ ranged from 0.93 to 1.00. The same research group [239] employed different classifiers such as logistic regression and QDA with features selected from 31 features by using stepwise feature selection based on the Akaike information criterion. Their system with logistic regression achieved an *A*_*z*_ value of 0.92 in distinction between 19 malignant and 16 benign nodules in thin-slice CE-CT.

Other LDC-based CAD systems include those developed by Way and colleagues [163]. They designed a system based on the morphological and texture features of pulmonary nodules on CT images, using a series of 96 lung nodules, with 44 biopsy-or-PET-scan-proven malignant nodules and 52 nodules that proved to be benign on biopsy or follow-up CT. The nodules were segmented using 3D active contours that were guided by a combination of 2D and 3D energies. Next, they extracted several morphological and texture-based features from the segmented nodules. The morphological features include volume, surface area, perimeter, maximum diameter, and maximum and minimum CT value inside the nodule. Using a stepwise method, they selected the most predictive features for use in the LDC. The classifier was trained and tested using a leave-one-out method, and the system achieved an *A*_*z*_ of 0.83. More recently, the same group [243] designed a system using the morphological features described above in combination with new measurements of the surface characteristics that quantified the smoothness and shape irregularity of the nodules. They calculated the ROC statistics for LDCs designed with and without the new surface feature, and found a significant (*P* < 0.05) improvement in performance with the *A*_*z*_ increasing from 0.821 to 0.857 in the classification of 124 malignant and 132 benign nodules in 152 patients. Aoyama et al. [236] used LDC for the distinction between malignant and benign nodules in thick-slice screening LDCT. They achieved an *A*_*z*_ value of 0.846 for a database of 73 patients with 76 primary cancers and 342 patients with 413 benign nodules.

One of the early neural network-based CAD systems was developed by Gurney and Swensen [351]. They compared two systems, one using a neural network-based classifier and one using a Bayesian classifier. Both systems used a combination of subjectively evaluated clinical and radiologic characteristics including border smoothness, spiculation, and lobulation. The Bayesian system showed a significantly (*P* < 0.05) higher level of performance (*A*_*z*_ = 0.894) than the neural network-based system (*A*_*z*_ = 0.871). Another neural network-based system using subjectively extracted features was developed by Matsuki et al. [234]. The radiological features included shape-based parameters such as border definition, spiculation, and concavity as well as other associated features such as blood vessel involvement, lymphadenopathy, and emphysematous changes. From a series of 155 nodules found on HRCT (99 malignant, 56 benign), features were extracted by attending radiologists using subjective rating scales and used to train the neural network. The neural network alone showed a high level of performance (*A*_*z*_ = 0.951) and significantly increased the radiologists' performance, increasing the *A*_*z*_ value from 0.831 to 0.959.

Other CAD systems have been designed to automatically define and extract features as well as classify nodules. For example, Henschke et al. [230] adapted the S-MODALS neural network, originally designed for tactical and strategic reconnaissance, to the task of nodule classification. Features were automatically selected from the example image using a NNs' clustering technique with operator-defined selection parameters including spatial separation of features and the degrees of similarity and dissimilarity that grouped features into clusters. The system was tested on a series of 28 biopsy-proven nodules (14 malignant, 14 benign), and all but 3 benign nodules were correctly classified. Another neural network system based on using automatically extracted features was designed by Lo et al. [235] and used a combination of radiographical parameters including vascularity, CT density distribution, and shape indices including aspect ratio, circularity, irregularity, extent, compactness, and convexity. Nodules were segmented using an automatic thresholding method, and the resulting 3D volumes were automatically smoothed and pruned of vasculature. The vascular index was calculated during this smoothing process, and shape indices were calculated from the resulting volume. Using a leave-one-out method, they trained the neural network on a series of 48 nodules (24 malignant, 24 benign). The results yielded an *A*_*z*_ value of 0.89, and they found that the most predictive features were the vascular index, size, compactness, and difference entropy of the CT density.

Suzuki et al. [117] developed a multiple MTANN scheme for the classification task based on training the MTANN classifier with a set of benign and malignant nodules. They achieved an *A*_*z*_ value of 0.88 for thick-slice screening LDCT scans of 73 patients with 76 primary cancers and 342 patients with 413 benign nodules. Chen et al. [244] employed ANN ensemble to classify 19 malignant and 13 benign nodules, and they achieved an *A*_*z*_ value of 0.915. Nakamura et al. [237] compared the performance of two separate networks, one trained on 8 subjective features rated by radiologists (i.e., nodule size, shape (round-to-elongated), marginal irregularity, spiculation, border definition, lobulation, and nodule density (contrast)) and the other trained on 12 matched features automatically extracted from chest radiographs (i.e., effective diameter, degree of circularity, degree of ellipticity, magnitude and coarseness of irregular edge patterns, mean gradient, radial gradient index, tangential gradient index, mean pixel, and and line enhancement index). Both sets employed shape-based features including margin irregularity, spiculation, lobulation, and nodule shape as well measures of homogeneity and CT density. The network based on objective features demonstrated the highest level of performance (*A*_*z*_ = 0.854) and was followed by the subjective feature network (*A*_*z*_ = 0.761) and then the radiologists (*A*_*z*_ = 0.752).

Iwano et al. [238] developed a system to automatically classify pulmonary nodules detected on HRCT into different shape categories and compared the performance to radiologists. The nodules were extracted from a series of 102 CT images without a prior diagnosis of malignancy and were classified into different shape categories based on quantitative measures of aspect ratio, circularity, and their second central moment. The results were compared to a subjective classification by radiologists, and they found that the automated system classified the nodules as accurately as the radiologists. Although no direct attempt at automatic diagnosis was carried out, they concluded that the system had the potential to aid radiologists in classifying nodules as malignant or benign based on the correlation between certain shape categories and the underlying pathology. The same research group [242] extended their work on 107 HRCT images and achieved a sensitivity of 76.9% and a specificity of 80% with their system based on LDA with two features (circularity and second moment) in the classification of a total of 52 malignant and 55 benign nodules.

Matsuoka et al. [240] analyzed the differences in nodule appearance on HRCT images from emphysematous and nonemphysematous patients based on subjective and quantitative measures of nodule appearance. Using a series of 41 emphysematous patients (21 malignant nodules, 20 benign nodules) and 40 non-emphysematous patients (20 malignant nodules, 20 benign nodules), two radiologists, who were blinded to the diagnosis, independently evaluated the appearance of the nodules and classified nodules as being either malignant or benign. The fractal dimensions of the nodule interfaces and circularity of the nodule shape were calculated and the percentage of the nodule surrounded by emphysema was obtained. In patients with emphysema, there were no significant differences in fractal dimension, circularity, spiculation, or frequency of lobulation between malignant and benign nodules. Of all the nodules found in patients with emphysema, 63% were correctly diagnosed. Thirteen benign nodules (65%) were misdiagnosed as malignant in patients with emphysema. Of the nodules in non-emphysematous lungs, 93% were correctly diagnosed. The mean percentage of the emphysematous tissue around the nodule was greater for misdiagnosed nodules than for correctly diagnosed nodules (*P* < 0.003), indicating that its presence complicates the diagnosis of pulmonary nodules. Lee et al. [245] developed a two-step supervised learning scheme based on a set of image-based gray-level, texture, and shape features combining a genetic algorithm with a random subspace method. They achieved an *A*_*z*_ value of 0.889 in classification between 62 malignant and 63 benign nodules.

Recently, El-Baz et al. [352, 353] proposed a 2D approach for early assessment of malignant lung nodules based on analyzing the spatial distribution of the Hounsfield values for the detected lung nodules. Spatial distribution of the Hounsfield values comprising the malignant nodule appearance was modeled with a 2D rotationally invariant second-order MGRF. To account for the whole 3D appearance of the lung nodules, they extended their approach in 3D to work on 3D lung nodule data [246, 354]. More recently, El-Baz et al. [247, 355–358] proposed an alternative, advanced method for diagnosing malignant lung nodules by their shapes. In this method, the 3D surfaces of the detected lung nodules are approximated by spherical harmonic analysis, which represented a 3D surface of the lung nodule supported by the unit sphere with a linear combination of special basis functions, called *spherical harmonics* (SHs). The lung nodule shape complexity was described with a new shape index, the estimated number of the SHs, which was used to distinguish between malignant and benign lung nodules.

 Thus, various approaches have been proposed in CADx systems. Database size varied in different studies; CT scans in the databases included screening LDCT, standard diagnostic CT, and HRCT. Studies on the development of CADx systems for the distinction between malignant and benign lung nodules in CT based on shape and appearance features are summarized in Table 9. In summary, the existing approaches that classify the lung nodules based on the extracting 2D features (e.g., round, lobulated, ragged, and halo) cannot consider the whole variability of lung nodules. Assessing the lung nodules using 3D metrics can enhance the classification accuracy. However, there is a need for developing qualitative measures that have the ability to describe the whole shape and appearance of the detected nodules. Another issue is that the existing set of shape and appearance features (e.g., curvature roundness) depend on the accuracy of the nodule segmentation algorithm. This makes a classification method, based on these features, difficult for clinical practitioners to use. Other investigators integrated the information from images captured using different types of image modalities (e.g., CT and PET) and investigated the impact of fusing the information obtained from these images on the accuracy of diagnosis. In the next section, we overview the related work done in this field.

### 5.3. PET/CT Nodule Diagnosis

 Since the combination of PET and CT information has shown an improvement in the delineation of lung nodule contours and the estimation of their volumes (see Section 4.3), PET/CT fusion has been widely considered in lung cancer applications such as the tumor staging and the pulmonary nodule diagnostics. In PET images, the malignant cells have unregulated metabolism that results in having higher FDG uptake that permits malignancy to be detected. Reported studies [319–324, 359, 360] used this characteristic to detect malignant *solitary pulmonary nodules* (SPNs) in PET. SPNs are single, spherical, well-circumscribed, radiographic opacity that measures ≤3 cm in diameter. Provided a visually validated diagnostics of the SPNs in PET images, these studies [319–324, 359, 360] have reported an SPN diagnostic accuracy with a sensitivity of 88–96% and a specificity of 70–90% for malignant cells (see Table 10 for more detail).

 Using PET alone without incorporation of CT was reported to provide imprecise information on the exact location of focal abnormalities [361] and can result in *false-negative* (FN) errors for lesions with low 18F-FDG uptake value [321, 362, 363] and FP errors in patients with active tuberculosis, histoplasmosis, and rheumatoid nodules. Annema [364] reported the FP findings of PET to be up to 39%, despite the high negative predictive value of PET, suggesting that the PET-positive *mediastinal lymph nodes* (MLN) were further biopsied in order to confirm or rule out metastasis.

To investigate the integration of PET and CT information on the accuracy of the malignancy detection, Nie et al. [327] developed an ANN approach based on CT alone, PET alone, and both CT and PET for distinguishing benign and malignant pulmonary nodules. Their results show that the accuracy of PET/CT (*A*_*z*_ = 0.95) is higher than that of the CT (*A*_*z*_ = 0.83) and the PET (*A*_*z*_ = 0.91). Nakamoto et al. [328] compared the diagnosis accuracy of CT, side-by-side PET/CT, and software-fused PET/CT. They documented that the software fusion of PET/CT resulted in the highest accuracy on patients with lung cancer. Keidar et al. [325] compared the diagnosis performance of PET/CT and PET alone. Using PET alone resulted in a higher FP error rate. A higher specificity was achieved using PET/CT suggesting that the anatomical information on CT is an independent crucial variable in determining malignancy. Yi et al. [326] investigated the sensitivity, specificity, and accuracy for predicting malignant nodules on helical dynamic CT and PET/CT. They documented that all malignant nodules were interpreted correctly using dynamic helical CT or PET/CT. Lardinois et al. [361] investigated tumor staging using PET/CT versus PET or CT alone. Their results showed that the PET/CT fusion is a trustworthy means of nodule diagnosis that has improved the accuracy of the tumor staging.

Table 11 summarizes the evaluation results of nodule malignancy in fused PET/CT systems. The experiments involved in these studies [325–328, 361] have shown that using PET/CT achieved a higher diagnostic power than CT or PET alone, suggesting that the PET/CT fusion may present an advancement in lung cancer applications.

## 6. Discussion and Conclusions

 Designing efficient CAD systems for lung cancer is very important since early diagnosis can improve the effectiveness of treatment and increase the patient's survival rate. In this paper, an overview of more than 360 articles that appeared in the field are presented to address the challenges and methodologies of the current CAD systems for lung cancer. This paper addresses the current approaches and their strengths and limitations, which were developed for each stage of lung cancer CAD systems, that is, for lung segmentation, lung nodule detection and segmentation, and lung nodule diagnosis. In the final section, we summarize this work by outlining the research challenges that face each stage in lung cancer CAD systems. In addition, the suggested trends to solve these challenges are presented.

### 6.1. Research Challenges

 Several challenges and aspects have been facing CAD systems for lung cancer. These challenges can be summarized as follows.

- To efficiently reduce the search space for lung nodules, accurate segmentation of the lung fields should be provided. The segmentation of lungs is challenging due to inhomogeneities in the lung region and pulmonary structures and of similar densities such as arteries, veins, bronchi, and bronchioles. Technical issues of the lung segmentation techniques should be further investigated. These technical issues include the automation level of the technique, the sensitivity of the method to the scanning parameters, the efficiency of an algorithm to work with different image modalities (e.g., CT, LDCT, or CE-CT), and the ability of the algorithm to provide a proper lung segmentation in cases with severe pathologies that are associated with inhomogeneities in the pathological lungs.
- Designing an efficient CADe system for detecting lung nodules is still challenging. Important factors should be investigated including the automation level, the speed, the ability to detect nodules of different shapes, for example, irregularly shape nodules not only the spherical ones, and the ability of the CADe system to detect cavity nodules, nodules attached to the lung borders, and small nodules (e.g., less than 3 mm).
- Several challenges and aspects have been facing lung nodule segmentation techniques, such as the ability of a technique to segment the challenging types of nodules, and the automation level of the technique and its robustness.
- Volumetric measurements of growth rate should take into account the global motion of the patients due to their movements and the local motions of the whole lung tissues due to breathing and heart beating. The application of global and local registration directly to the segmented nodule leads to the inability to discriminate between the changes due to the true growth of the lung nodules and the changes in the nodule shape which come from breathing and heart beating. These challenging factors should be further investigated.
- Special types of lung nodules such as cavities and ground glass nodules can not be diagnosed using the current growth rate techniques, so further methods and nodule descriptors are needed for diagnosing these nodules.
- The existing set of shape and appearance features (e.g., curvature, and roundness) depend on the accuracy of the nodule segmentation algorithm. This makes a classification method, based on these features, difficult for clinical practitioners to use. So, there is a need for developing qualitative measures that have the ability to describe the whole shape and appearance of the detected nodules.
- Larger databases for efficient validation of the proposed approaches should be provided.

### 6.2. Trends

To address the aforementioned challenges, recent trends for lung cancer CAD systems involve the following aspects.

- For accurate volumetric growth rate measurements, a recent trend applies global and local registration to the lung fields instead of the segmented nodule in order to discriminate between the changes due to the true growth of the lung nodules and the changes in the nodule shape which come from breathing and heart beat [208, 339–346].
- More powerful, sophisticated shape and appearance features for lung nodule detection and diagnosis need further investigations. A recent trend models the spatial distribution of the Hounsfield values of the detected lung nodules with the *Markov Gibbs random field* (MGRF) models in order to accurately describe the nodule appearance [246, 352–354]. Another trend describes the lung nodule's shape by representing its 3D surface with a linear combination of *spherical harmonics* (SH) [247, 355–358]. The recent works suggested employing different types of appearance and shape features to achieve better detection and diagnosis of lung nodules.
- Investigations of using the microfluidics technology to mimic the contractions and expansions of the lung and nodules during normal breathing have recently been explored to provide more realistic phantoms in order to validate the volumetric growth rate measurements [305–307].
- Investigators integrated the information from images captured using different types of image modalities (e.g., CT and PET) and investigated the impact of fusing the information obtained from these images on the accuracy of diagnosis. The experiments involved in this survey showed that using PET/CT achieved a higher diagnostic power than CT or PET alone, suggesting that the PET/CT fusion may present an advancement in lung cancer applications. Still, important points need further investigations, such as the poor resolution of PET, the exact definition of tumor edges, and the misregistration between PET and CT images.

The clinical importance of the diagnosis of lung cancer has been reflected over more than 360 publications presented in this survey. The presented challenges and trends, in this section, suggested that investigating more efficient CAD systems for lung cancer will remain a very active research area and suggested that more comprehensive studies are necessary for establishing the state-of-the-art CAD systems in this active research field.

## Figures and Tables

**Table 1:** Review of current approaches for lung segmentation. Studies are ordered by their publication year.

Study	Database	Dim	Image size	Approach	AL	Running time	GS	Performance
Hu et al. [3]	24 datasets from 8 subjects	3D	512 × 512, 3 mm thin	Iterative threshold, dynamic programing, morphological operations	A	2-3 min on a 300 MHz processor, 512 MB RAM (512 × 512 × 120)	229 manual traced images	RmsD = 0.54 mm (0.8 pixel)

Mendonca et al. [43]	47 image radiographs	2D	NA	Spatial edge detector	A	NA	47 manual traced data	Sen. = 0.9225, PPV = 0.968

Yim et al. [8]	10 subjects	3D	512 × 512, 0.75–2 mm thin	Region growing, connected component	A	42.3 sec on a 2.5 GHz processor, 2.0 GB RAM (512 × 512 × 352)	10 manual traced data	RmsD = 1.2 pixel

Sluimer et al. [30]	26 scans	3D	512 × 512, 0.75–2.0 mm	Shape-based	A	3 hr on a 2.8 GHz processor, 2.0 GB RAM (512 × 512 × 400)	10 manual traced data, each 4 slice	OM = 0.8165, AD = 1.48 mm, HD = 13.45 mm

Campadelli et al. [42]	487 image radiographs	2D	256 × 256	Spatial edge detector	A	NA	487 manual traced data	Sen. = 0.9174, Spec. = 0.9584, PPV = 0.9197, Accu. = 0.9437

Korfiatis et al. [44]	23 scans	3D	512 × 512	Wavelet edge detector	A	3 min on a 2.8 GHz processor, 2 GB RAM (512 × 512 × 50)	22 manual traced data	OM = 0.983, AD = 0.77 mm, RmsD = 0.52 mm

Gao et al. [13]	8 subjects	2D	512 × 512 × 240	Thresholding	A	15–20 min on a 3.0 GHz processor, 1 GB RAM (512 × 512 × 240)	8 manual traced datasets	DSC = 0.9946

Silveira et al. [18]	1 subject	2D	512 × 512, 1 mm thin	Deformable model	A	NA	NA	Qualitative assessment

Pu et al. [12]	20 datasets	2D	512 × 512, 1.25 mm thin	Thresholding	A	1 min on a 2.11 GHz processor, 2 GB RAM (512 × 512 × 540)	20 manual traced datasets	FP/GT = 0.43%, FN/GT = 1.63%

Shi et al. [22]	247 image radiographs	2D	256 × 256	Shape-based deformable model	A	75 sec per image on a 3 GHz processor, 1 GB RAM (512 × 512)	247 manual traced images	OM = 0.92, AD = 1.78 pixel

El-Baz et al. [35, 36]	10 image datasets	3D	512 × 512 × 182, 2.5 mm thin	Statistical MGRF model	A	1.65 sec per image on a 3.2 GHz processor, 16 GB RAM	1820 manual traced images	Accu. = 0.968

Annangi et al. [19]	1130 image radiographs	2D	128 × 128 and 256 × 256	Shape-based deformable model	A	7 sec per image on a 2.4 GHz processor	1130 manully traced images	DSC = 0.88

Kockelkorn et al. [32]	22 scans	3D	0.9-1.0 mm	Prior training, statistical classifier	UI	10 min	12 manual traced data	OM = 0.96, AD = 1.68 mm

Besbes and Paragios [28]	247 image radiographs	2D	256 × 256, 1 mm thin	Shape-based	A	NA	123 manual traced data	OM = 0.9474, AD = 1.39 pixel

Sofka et al. [31]	260 scans	3D	0.5–5.0 mm	Shape-based	A	NA	68 manual traced data	SCD = 1.95

Hua et al. [33]	15 scans	3D	0.3–0.9 mm	Graph-search	A	6 min on a 2.0 GHz processor, 32 GB RAM	12 semiautomated traced data	HD = 13.3 pixel, Sen. = 0.986, Spec. = 0.995

Sun et al. [26]	30 scans	3D	512 × 512 × 424–642, 0.6–0.7 mm thin	Shape-based	A	6 min per dataset on a NVIDIA Tesla C1060 processor (240 thread), 4 GB RAM	30 manually corrected traced data	DSC = 0.975, AD = 0.84 mm, SPD = 0.59 mm, HD = 20.13 mm

Abdollahi et al. [39, 40]	11 scans	3D	512 × 512 × 390, 2.5 mm thin	Statistical MGRF model	A	NA	11 manual traced data	DSC = 0.960

AL denotes automation level (A: automatic, UI: user interactive; Dim denotes the approach dimension (2D or 3D).

GS stands for gold standard; NA stands for non applicable.

DSC denotes the Dice similarity coefficient; DSC = 2TP/(2TP + FP + FN); Accu. denotes the accuracy, Accu. = (TP + TN)/(TP + TN + FP + FN).

OM denotes overlap measure, OM = TP/(TP + FP + FN); Sen. denotes the sensitivity, Sen. = TP/(TP + FN).

Spec. denotes the specificity, Spec. = TN/(TN + FP); PPV denotes positive predictive value, PPV = TP/(TP + FP).

RmsD denotes the root mean square difference of the distance between the segmentation and the ground truth.

AD denotes the mean absolute surface distance.

HD denotes the Hausdorff distance, the mean maximum distance of a set to the nearest point in the other set.

SPD denotes the mean signed border positioning error.

SCD denotes symmetrical point-to-mesh comparison error.

**Table 2:** Classification component in CADe systems. Studies are ordered by their publication year.

Study	Feature/input	Classifier/method	Database	Performance
Giger et al. [123]	Geometric features	Comparison of geometric features	Thick-slice diagnostic CT scans of 8 patients with 47 nodules	Sensitivity of 94% with1.25 FPs per case

Kanazawa et al. [81]	8 features	Rule based scheme	Helical CT scans from 450 patients with 230 nodules (a total of 15,750 image sections)	Sensitivity of 90%

Armato et al. [9, 124]	Nine 2D and 3D features	Rule-based scheme and LDA	Thick-slice (10 mm) diagnostic CT scans of 43 patients with 171 nodules	Sensitivity of 70% with 42.2 FPs per case in a leave-one-out test

Lee et al. [71]	13 features	Rule-based scheme and LDA	Thick-slice (10 mm) diagnostic CT scans of 20 patients with 98 nodules	Sensitivity of 72% with 30.6 FPs per case

Ko and Betke [64]	Location and 2 shape features (circularity and roundness)	Rule-based scheme	Helical CT scans of 16 studies (8 initial and 8 followup) obtained from 8 patients with 370 nodules	Sensitivity of 86%

Brown et al. [87]	Prior models based on 4 features	Fuzzy matching	Thick slice (5–10 mm) CT scans of 17 patients with 36 nodules	Sensitivity of 86% and 11 FPs per case

Wiemker et al. [72]	4 shape and intensity features	NA	Thin-slice (1 mm) HRCT scans of 50 subjects (a total of more than 20,000 image sections); 12 scans were reviewed by radiologist with 203 nodules	Sensitivity of 86% with 4.9 FPs per case for nodules with diameter ≥1 mm and sensitivity of 95% with 4.4 FPs per case with diameters ≥2 mm

Gurcan et al. [78]	Six 2D and 3D features	Rule-based scheme and LDA	Thick-slice (2.5–5 mm, mostly 5 mm) diagnostic CT scans of 34 patients with 63 nodules	Sensitivity of 84% with 74.4 FPs per case in a leave-one-out test

Suzuki et al. [111]	Pixel values in a 9 × 9 subregion	Multiple MTANNs	Thick-slice (10 mm) screening LDCT scans of 63 patients with 71 nodules with solid, partially solid, and nonsolid patterns, including 66 cancers	Sensitivity of 80.3% with 4.8 FPs per case in a validation test

Mekada et al. [63]	Minimum directional difference filter	Rule-based scheme	CT scans of 6 subjects with 361 nodules (160–350 sections per case)	Sensitivity of 71% and 7.4 FPs per case

Arimura et al. [116]	Pixel values in a 9 × 9 subregion for MTANNs (selected features for LDA)	Rule-based scheme followed by multiple MTANNs (or LDA with Wilks' lambda stepwise feature selection)	106 thick-slice (10 mm) screening LDCT scans of 73 patients with 109 cancers with solid, partially solid, and nonsolid patterns	Sensitivity of 83% with 5.8 FPs per case in a validation test (or a leave-one-out test for LDA)

Awai et al. [74]	6 geometric features	Artificial neural network classier	CT scans of 82 patients with 78 nodules (a total of 3,556 image sections)	Sensitivity of 80% with 0.87 FPs per section

Paik et al. [69]	SNO method that describes the shape and geometry	Rule-based scheme	CT scans of 8 patients	Sensitivity of 90% with 5.6 FPs per case in a cross validation test

Farag et al. [125, 126]	NA	Template modeling approach using LS	Thin-slice (2.5 mm) screening LDCT scans of 16 patients with 119 nodules and 34 normal patients	Sensitivity of 93% with 3.4 FPs per case

Ge et al. [127]	44 features including 3D gradient field descriptors and ellipsoid features	LDA with Wilks' lambda stepwise feature selection	82 thin-slice (1.0–2.5 mm) CT scans of 56 patients with 116 solid nodules	Sensitivity of 80% with 14.7 FPs per case in a leave-one-out test

Mendonca et al. [70]	Geometric and intensity models combined with eigen curvature analysis	Rule-based scheme	Thin-slice (1.25 and 2.5 mm) CT scans of 242 exams from two institutions: 50 CT scans with 109 nodule and 192 CT scans with 210 nodules	Sensitivity of 67.5% and 9.3 FPs per case for data from the first 50 CT scans and sensitivity of 62.9% and 10.3 FPs per case for the second 192 CT scans in a leave-one-out test

Matsumoto et al. [128]	8 features	LDA	Thick-slice (5 or 7 mm) diagnostic CT scans of 5 patients (4 of which used contrast media) with 50 nodules	Sensitivity of 90% with 64.1 FPs per case in a leave-one-out test

Yuan et al. [129]	NA	ImageChecker CT LN-1000 by R2 Technology	Thin-slice (1.25 mm) CT scans of 150 patients with 628 nodules	Sensitivity of 73% with 3.2 FPs per case in an independent test

Pu et al. [130]	NA	Scoring method based on the similarity distance combined with a marching cube algorithm	Thin-slice (2.5 mm) screening CT scans of 52 patients with 184 nodules including 16 nonsolid nodules	Sensitivity of 81.5% with 6.5 FPs per case

Retico et al. [131]	Pixel values in a subvolume	Voxel-based neural approach (MTANN)	Thin-slice (1 mm) screening CT scans of 39 patients with 102 nodules	Sensitivities of 80–85% with 10–13 FPs per case

Ye et al. [15]	15 features	Rule-based scheme followed by a weighted SVM	Thin-slice (1 mm) screening CT scans of 54 patients with 118 nodules including 17 non-solid nodules	Sensitivity of 90.2% with 8.2 FPs per case in an independent test

Golosio et al. [132]	42 features from multithreshold ROI	Fixed-topology ANN	Thin-slice (1.5–3.0 mm) CT scans of 83 patients with 148 nodules that one radiologist detected from the LIDC database	Sensitivity of 79% with 4 FPs per case in an independent test

Murphy et al. [133]	Features selected from 135 features	KNN	Thin-slice screening CT scans of 813 patients with 1,525 nodules	Sensitivity of 80% with 4.2 FPs per case in an independent test

Messay et al. [134]	Features selected from 245 features	LDA and quadratic discriminant analysis with feature selection	Thin-slice CT scans of 84 patients with 143 nodules from the LIDC database	Sensitivity of 83% with 3 FPs per case in a 7-fold cross-validation test

Tan et al. [135]	45 features	Feature-selective classifier based on a genetic algorithm and ANNs	Thin-slice CT scans of 125 patients with 80 nodules that 4 radiologists agreed from the LIDC database	Sensitivity of 87.5% with 4 FPs per case in an independent test

Riccardi et al. [136]	Maximum intensity projection data from the volume of interest	Heuristic approach (rule-based scheme) and SVM	Thin-slice CT scans of 154 patients with 117 nodules that 4 radiologists agreed on from the LIDC database	Sensitivity of 71% with 6.5 FPs per case in a 2-fold cross-validation test

**Table 3:** Studies on volumetric nodule segmentation reported from 1998 to 2005. Studies are ordered by their publication year. The purpose, type, and basic idea of each reported segmentation method are briefly described. Data and method used for validation of the proposed methods are also described.

Study	Purpose	Type	Method	Database	Validation and performance
Kawata et al. [137, 138]	Solitary, solid	Deformable model, 3D	Geometric deformation flow of 3D LS surface proposed by Caselles et al. [139]	62 nodules (47 malignant 15 benign) between 6 and 25 mm	Qualitative: correct segmentation of nodules with ill-defined surface; malignancy classification with two 3D surface characteristics

Yankelevitz et al. [140, 141]	Small, solitary, solid	Threshold (2D [141]/3D [140])	*k*-means segmentation for automatic threshold estimation	Phantom (3.20 and 3.96 mm); in vivo: 13–15 nodules in repeat CTs	RMS error in volume measurement: ±3% (3D); volumetry: effective measurement of malignant growth of nodules as small as 5 mm (2D) with doubling time less than 177 days (3D)

Zhao et al. [142, 143] and Wiemker and Zwartkruis [144]	Small, juxtavascular	Threshold (2D [142]/3D [143])	Multicriterion automatic threshold estimation with average gradients along lesion contour and with boundary shape compactness, lesion segmentation by CCL and MOs, efficient average gradient computation [144]	9 nodules (<10 mm) with manual GT (2D)	Mean difference of 0.97 pixels was not statistically significant: *P* = 0.90 (2D)

Xu et al. [145]	Juxtavascular, juxtapleural, calcification	Dynamic programing, 2D	2D contour optimization by DP. Calcification removal by EM classification of air, soft and calcified tissues. Semiautomatic contour correction by observers	4 nodules	Qualitative discussion only

Fetita et al. [76]	Juxtavascular, juxtapleural	Automatic, mathematical morphology	Gray-level MO with SMDC-connection cost. Juxtavascular cases by morphological dilation. Juxtapleural cases by global lung segmentation	300 nodules with 2–20 mm diameters of 10 patients	Detection performance: 98% sensitivity and 97% specificity for isolated and juxtavascular nodules; 90% sensitivity and 87% specificity for juxtapleural nodules

Ko et al. [146]	Small, solid/GGO	Threshold	Two-value thresholding with partial-volume correction based on CT intensity values	Phantom: 40 synthetic nodules (<5 mm, 20 solid and 20 GGO)	Average error in volume measurement: 2.1 mm^3^

Kostis et al. [73, 147]	Small, juxtapleural, juxtavascular	Mathematical morphology	Isotropic resampling for partial-volume effect; binary segmentation by thresholding and CCL followed by vascular subtraction and pleural surface removal with iterative MOs	105 small nodules (<10 mm) of two time-points	Success rate: 80% for 21 juxtavascular cases; reproducibility study in measuring the percentage volume changes [147]

Okada et al. [148–151]	Small, juxtavascular, GGO	Robust anisotropic Gaussian fitting and mean shift (MS)	Robust anisotropic Gaussian intensity model fitting with MS segmentation in 4D spatiointensity domain	77 nodules of 3–25 mm diameters of 14 patients	Success rate: 89.6%; consistency: 1.12 voxel mean error for lesion center estimate when perturbing initialization

Kuhnigk et al. [152, 153]	Small, juxtavascular, juxtapleural	Automatic, region growing, and mathematical morphology	Region growing and CCL for initial segmentation. Juxtapleural and juxtavascular cases by convex hull and MOs. Volume estimation with partial-volume effect handling	Phantom: 31 nodules of various types; in vivo: 105 nodules with diameter larger than 4.6 mm of 16 patients	Success rate: 91.4%; inter-observer variability: 0.1% median error and 7.1% error at 95% limit; inter-scan variability: 4.7% median error and 26.9% error at 95% limit; volumetry median error with phantom: −3.1% for vascularized cases; −10.2% for juxtapleural cases

Mullally et al. [154]	Solitary, solid	Automatic, threshold	Automating the selection of VOI for thresholding-based segmentation methods by Zhao et al. [142, 143] and Ko et al. [146]	Phantom: 40 nodules (2.4 and 4.9 mm); in-vivo: 29 nodules in repeat CTs; manual GTs by a radiologist	Volume accuracy: 43% error for phantoms; 50% error for in vivo nodules

Shen et al. [155]	Juxtapleural	Surface analysis	Lung surface removal for juxtapleural nodule segmentation by local surface smoothing	20 juxtapleural nodules of a patient	Average RMS deviation from median by various click points: <2% except for one case; volumetry consistency: 60% of all varying click points leads to the same volume measure

Zhang et al. [97, 156]	GGO, juxtavascular	Probabilistic classification	MAP segmentation with a conditional distribution by a two-class GMM and with a priori by MRF. MAP optimization solved by iterated conditional modes. Juxtavascular cases by vessel segmentation. Conditional distribution adapted to each nodule to account for intensity offsets [156]	23 GGO nodules of 8 patients; manual GTs by two radiologists [156]	Success rate: 91.3%; consistency with 3 different clicks: 0.96 ± 0.02 overlaps for all 21 successfully segmented cases; average overlap with GTs: 0.69 ± 0.05; interobserver consistency: 0.73 ± 0.04

Okada et al. [157] Okada et al. [158, 159]	Small, juxtavascular, GGO, juxtapleural	Probabilistic classification [157] and mathematical morphology [158, 159]	Likelihood ratio test in spatiointensity joint domain after robust anisotropic Gaussian fitting by [151]. Juxtapleural cases by morphological opening and by prior constrained MS for rib bone suppression	1312 nodules of 39 patients; 123 true-negative cases included 108 juxtapleural cases	Success rate: 83.5% by [157]; 94.8% by [158, 159] overall; 71.5% for the juxtapleural/true-negative cases

GGO: ground-glass opacity (nonsolid and partially solid) nodules; LS: level sets; DP: dynamic programing; MO: morphological operations; CCL: connected-component labeling; EM: expectation-maximization; MAP: maximum a posteriori; MRF: Markov's random fields; KNN: *k*-nearest neighbor; GMM: Gaussian mixture model; LDA: linear discriminant analysis; GT: segmentation ground truth.

**Table 4:** Studies on volumetric nodule segmentation reported from 2006 to 2010. See Table 3 for description of captions.

Study	Purpose	Type	Method	Database	Performance
El-Baz et al. [160] Farag et al. [161]	General, cavity	Deformable model, 3D	Lesion boundary optimization by fitting a prior model with MRF and an appearance model with a bimodal LCDG	350 nodules with 3 to 30 mm of 29 patients; manual GTs by a radiologist	Segmentation error: min. 0.4%, max. 2.25%, mean 0.96%

van Ginneken [162]	General	Discriminative classification	Soft segmentation by supervised classifier. KNN regression of voxel-wise nodule probability with intensity features (gradient magnitude, Hessian eigenvalues, etc., over Gaussian scale-space)	LIDC1 dataset: 23 nodules with manual GTs	Average soft-overlap: 0.66 ± 0.18 (0.52 ± 0.25 by [73]); average percentage volume error: 23.7% ± 124.5% (by 510.8% ± 1577.7% [73])

Way et al. [163, 164]	General	Deformable model, 2D/3D	Successive 2D active contour with 3D gradient, 3D curvature, and mask energy terms with greedy optimization	LIDC1 dataset: 23 nodules with manual GTs	Average overlap: ranging between 0.07 and 0.63 across varying probabilistic GTs; median percentage volume error: 40%

Goodman et al. [165]	Juxtavascular	Watersheds	Watersheds segmentation followed by a model-based shape analysis to handle juxtapositions	50 nodules of 25 patients (<20 mm) with 17 irregular/spiculated margins, 16 juxtapleural, 10 juxtavascular, and 2 GGO cases	Success rate: 97% over 450 measures (3 time-points by 3 observers)

Zhou et al. [166, 167]	GGO, juxtavascular	Probabilistic classification	Voxel-wise classification by comparing a nonparametric kernel density estimate of GGO intensity model with that of each local neighborhood by the Bhattacharya distance. Juxtavascular cases by eigen analysis of Hessian	10 GGO nodules	Only qualitative assessment

Yoo et al. [168]	GGO, juxtavascular	Deformable model, 3D	Asymmetric 3-phase deformable model of two LS functions	3 nodules	Only qualitative assessment

Wang et al. [169]	General	Dynamic programming, 3D	Transformation of 3D image to 2D polar-coordinate image by spiral scanning followed by 2D contour optimization by DP	LIDC1 dataset: 23 nodules with 4.0 to 33.6 mm diameter; LIDC2 dataset: 73 nodules with 3.8 to 30.2 mm diameter	Average overlap (LIDC1): 0.66 in [0.47, 0.89]; average overlap (LIDC2): 0.64 in [0.39, 0.87]

Nie et al. [170]	General	MS, 2D	MS clustering on a feature domain of convergence index by [171]	39 nodules with manual GTs	Average overlap: 0.83

Zheng et al. [172] Zheng et al. [173]	General	Graph-cut, coupled segmentation-registration, 2D, automatic	2D graph-cut segmentation coupled with B-spline nonrigid lung registration [172]. Spatially coherent segmentation by solving MRF with graph-cut [173]	12 nodules with manual GTs	Mean percentage of the nodule volume variation: 0.8 ± 0.6 in [172]

Browder et al. [174]	GGO, small, juxtavascular	Probabilistic classification	3-class (solid, non-solid, parenchyma) voxel-wise probabilistic classification with Gaussian intensity model. Bilateral filter by Tomasi used for noise removal. Juxtavascular cases by vessel removal filtering	ELCAP dataset: 75 cases with 5.6–17.5 mm in diameter; manual GTS by radiologists	Median growth consistency by geometric closeness metric: 1.87 (3.12 by radiologists)

Dehmeshki et al. [175]	Juxtavascular, juxtapleural	Region growing	Sphericity-oriented contrast-based region growing from an optimum seed point within a fuzzy connectivity map	815 nodules with 5–30 mm in diameter, 98 juxtapleural or juxtavascular cases	Success rate: 85–83%

Diciotti et al. [176]	Small, juxtavascular,	Semiautomatic, region growing	Target detection by LoG filtering followed by user selection. 3D region growing segmentation using a fusion-segregation criteria with geodesic distance	Phantom: 60 solid, juxtavascular, non-solid cases with 5.3–11 mm in diameter; ITALUNG dataset: 98 nodules; LIDC1 dataset: 23 nodules	Success rate: 86.3% (ITALUNG: 79.7% for juxtavascular); 83.3% (LIDC1: 75% for juxtavascular); volumetry RMS error: 1.0–6.6%

Kubota et al. [177, 178]	Small, juxtapleural, juxtavascular, solid, GGO	Region growing	Nodule enhancement by coupled competition-diffusion filtering. nodule core estimated as the maximum component of Euclidean distance map. Juxtapleural cases by estimating region core with centricity map. Segmentation and nodule extraction by region growing followed by convex hull	LIDC1: 23 nodules; LIDC2: 82 nodules; 820 nodules with manual diameter GTs	Average overlap (LIDC1): 0.69 ± 0.18 (0.67 ± 0.22 by [153], 0.57 ± 0.20 by [73], 0.52 ± 0.25 by [157]); average overlap (LIDC2): 0.59 ± 0.19 (0.56 ± 0.18 by [153], 0.45 ± 0.21 by [157])

Zheng et al. [179]	GGO	Opacity map estimation, 2D	Thresholding opacity map estimated by solving a linear equations system constructed with the graph Laplacian	40 slices of 11 patients; manual GTs	Average shortest distance along contours: 4.2 ± 4.9 pixels

Wang et al. [180]	General	Dynamic programming, 3D	Multidirection segmentation fusion by sequential dynamic 2D contouring ([145, 169]) applied to three orthogonal directions of a volume	LIDC1: 23 nodules for training; LIDC2: 64 nodules for testing	Overlap (LIDC1): mean 0.66, true-positive rate (TPR): 75%, false-positive rate (FPR): 15%; overlap (LIDC2): mean 0.58, true-positive rate (TPR): 71%, false-positive rate (FPR): 22%

Tao et al. [181]	GGO	Probabilistic classification	GGO nodule class-conditional probability map derived by an iterative LDA with GMMs of various intensity features. Nodule segmentation by applying shape-prior probability mask	1100 nodules with 100 GGO nodules; 60 cases with manual GTs	Average overlap: 0.68; voxel-wise classification success rate: 92.28% overall; 89.87% GGO

**Table 5:** Studies on volumetric nodule segmentation reported from 2011 to present. See Table 3 for description of captions.

Study	Purpose	Type	Method	Database	Validation and Performance
Farag et al. [182]	Juxtapleural	Deformable model, 3D	Variational LS segmentation with narrow band implementation	ELCAP database: 397 nodules of 50 patients, 115 juxtapleural cases	Success rate: 70% for juxtapleural cases

Zinoveva et al. [183]	General	Discriminative classification	Soft segmentation. CART decision-tree classifier trained with texture and intensity features. VI trimming after processing	LIDC2 dataset: 39 nodules with 3–30 mm in diameter; manual GTs by 4 radiologists	Median soft-overlap: 0.49 and 0.52 with VI trimming

Jirapatnakul et al. [184]	Juxtapleural	Surface analysis	Robust estimation of the pleural surface, surface removal by change detection over the estimated surface	150 solid juxtapleural nodules	Success rate: 98.0% (81.3% by [185])

Diciotti et al. [186]	Juxtavascular	Shape analysis	Refine an initial rough segmentation based on a local shape analysis on 3D geodesic distance map representations	ITALUNG dataset: 256 small nodules; LIDC12 datasets: 157 small nodules	Success rate: 84.8% (ITALUNG) and 88.5% (LIDC12) for automatic; 91.0% (ITALUNG) and 91.7% (LIDC12) for interactive mode

**Table 6:** Summary of lung nodule segmentation approaches from PET images. For each study, the table summarizes the number of the patients enrolled in the study and the type of the nodule delineation approach with respect to the methodology, the approach dimension, and the automation level.

Study	Patients	Delineation approach	Dim	AL
Kiffer et al. [187]	15	Coregistration	2D	A
Munley et al. [188]	35	Manual registration	NA	NA
Nestle et al. [189]	34	Visual	NA	NA
Mah et al. [190]	30	Thresholding	3D	A
Erdi et al. [191]	11	Thresholding	NA	A
Bradley et al. [192]	26	Thresholding	2D	A
Deniaud-Alexandre et al. [193]	101	Visual	NA	NA
Van Der Wel et al. [194]	21	Visual	NA	NA
Ashamalla et al. [195]	19	Thresholding	NA	A
Hatt et al. [196]	NA	Fuzzy hidden Markov chain	NA	A
Hatt et al. [197]	NA	Fuzzy classification	3D	A
Avazpour et al. [198]	11	Region growing	2D	A
Hatt et al. [199]	38	Fuzzy classification	NA	A

*Note that Dim denotes the approach dimension (2D, 3D), AL denotes automation level (A: automatic, UI: user interactive), and NA stands for nonapplicable.

**Table 7:** Assessing the effect of PET/CT on GTV. For each study, the number of patients, the PET/CT fusion method, and the increase and decrease in the GTV as a percentage of the total number of the study cases are reported.

Study	Patients	PET/CT fusion method	GTV increase	GTV decrease
Kiffer et al. [187]	15	Graphical co-registration	27%	NA
Munley et al. [188]	35	Visual	34%	NA
Nestle et al. [189]	34	Visual (side by side)	9%	26%
Mah et al. [190]	30	Software	22%	NA
Erdi et al. [191]	11	Software	64%	36%
Bradley et al. [192]	26	Software	46%	12%
Deniaud-Alexandre et al. [193]	101	Software	26%	23%
Ashamalla et al. [195]	19	Hardware	26%	26%
Van Der Wel et al. [194]	21	Visual	14%	52%
Avazpour et al. [198]	11	Software	NA	NA

**Table 8:** Growth-rate-based methodologies for following up pulmonary nodules.

Study	Purpose	Method	Database	Observations
Yankelevitz et al. [141]	To assess using early CT repeat to determine nodule growth rate	2D *growth rate* (GR) technique by measuring the maximal diameter of nodule	Repeat CT for 15 patients (15 nodules: 9 malignant, 6 benign (5–20 mm)) + spherical phantoms of known diameters	A single repeat after 30 days detects as small as 5 mm malignant nodule; all the 15 nodules are correctly classified

Yankelevitz et al. [140]	To determine the accuracy of CT volumetric measurements of small pulmonary nodules to assess growth and malignancy status	Exponential growth model to estimate the doubling time	13 patients (nodule <10 mm) (5 malignant and 8 benign) + synthetic phantoms of spherical, deformable, and different shape and sizes	(a) The synthetic nodule studies revealed that the volume could be measured accurately to within 3%. (b) All five malignant nodules grew, and all had doubling times less than 177 days. (c) All eight benign nodules had doubling times of 396 days or greater or showed a decrease in volume

Winer-Muram et al. [200]	To determine the range of growth rates of stage I lung cancers prior to treatment.	Volumetric measurement	50 patients, 50 tumor	Comparison of tumor volumes at serial CT examinations reveals a very wide range of growth rates. Some tumors grow so slowly that biopsy is required to prove that they are malignant

Borghesi et al. [201]	To evaluate the accuracy of software-calculated growth rate of small nodules in detecting malignancy	Volume doubling time was calculated on the Aquarius Workstation (TeraRecon, Inc.) with the *segmentation analysis and tracking* (SAT) module	29 patients (40 nodules (solid or noncalcified) 4–15 mm, glass opacities nodules were discarded); 4 of the nodules are given their diagnosis (3 benign and 1 malignant)	4 nodules are correctly classified

Wormanns et al. [202]	To assess the measurement precision of a software tool for volumetric analysis of nodules from two consecutive low-dose CT scans	Volumetric measurement	10 subject, 151 nodules	Taking into account all 151 nodules, 95% limits of agreement were −20.4 to 21.9% (standard error 1.5%)

Revel et al. [203]	To evaluate software designed to calculate pulmonary nodule volume in 3D	Volumetric measurement	54 nodules, 22 diagnosed: 13 benign and 9 malignant	Software measurement error of 6.38% of the previous volume measurement

Kostis et al. [147]	To determine the reproducibility of volume measurements of small pulmonary nodules on CT scans and to estimate critical time to follow-up CT	*Percentage volume change* (PVC) and *monthly volumetric growth index* (MVGI) were computed for each nodule pair	115 nodule	Factors that affect reproducibility of nodule volume measurements and critical time to follow-up CT include nodule size at detection, type of scan (baseline or repeat) on which the nodule is detected, and presence of patient-induced artifacts

Goo et al. [204]	To evaluate the effect of CT parameters and nodule segmentation thresholds on accuracy of volumetric measurement of synthetic lung nodules	Volumetric measurement	4 types of lung phantoms	For accurate measurement of the lung nodule volume, it is critical to select a section thickness and/or segmentation threshold appropriate for the size of a nodule

Reeves et al. [185]	To develop a method for measuring the change in nodule size from 2 CT image scans recorded at different times to establish the growth rate	Registration, adaptive thresholding, and knowledge-based shape matching	50 benign or 2YNC nodule	The variance in percent volume change was reduced from 11.54% to 9.35% through the use of registration, adaptive thresholding, and knowledge-based shape matching

Jennings et al. [205]	To retrospectively determine the distribution of stage I lung cancer growth rates with serial volumetric CT	Volumetric measurement	149 patients	At serial volumetric CT measurements, there was wide variability in growth rates. Some biopsy-proved cancers decreased in volume between examinations

Zheng et al. [172]	To simultaneously segment and register lung and tumor in serial CT data	Nonrigid transformation on lung deformation and rigid structure on the tumor	6 volumes of 3 patients, 83 nodules	The mean error of boundary distances between automatic segmented boundaries of lung tumors and manual segmentation is 3.50 pixels. The mean and variance of percentages of the nodule volume variations caused by errors in segmentation are 0.8 and 0.6

Jirapatnakul et al. [206]	To measures the nodule growth without explicit segmentation	*Growth analysis from density* (GAD) method to measure the growth rate	20 cases each with single nodule with scans several minutes apart (expected zero volume change), 38 cases with a stable nodule, 19 cases with a malignant nodule, and 4 malignant nodules with a complex appearance	Accuracy achieved was 37/38 for the stable benign nodules, 18/19 for the malignant nodules, and 4/4 for the complex malignant nodules

Marchianò et al. [207]	To assess in vivo volumetric repeatability of an automated algorithm for volume estimation.	Semiautomatic volumetric measurement	101 subjects, 233 nodules	The 95% confidence interval for difference in measured volumes was in the range of ±27%. About 70% of measurements had a relative difference in nodule volume of less than 10%

El-Baz et al. [208]	To monitor the development of lung nodules	Global and local registration, GR volumetric measurement	135 LDCT from 27 subjects, 27 nodules	All the 27 nodules are correctly classified based on GR measurements over 12 months

**Table 9:** Classification between malignant (**M**) and benign (**B**) nodules based on shape and appearance features.

Study	Purpose	Method	Database	Observations
Kawata et al. [227]	To characterize morphology of small pulmonary nodules	Using surface curvatures and a ridge line	Thin-section CT images for 56 cases including 42 **M** and 14 **B** nodules	The distribution of the nodule characteristics in the feature space shows good evidence of separation between the two classes

Henschke et al. [230]	To explore the usefulness of *neural networks* (NNs) to help in this differentiation	*Statistical-multiple-object detection and location system* (S-MODALS) NNs technique developed for *automatic target recognition* (ATR)	CT images of 28 pulmonary nodules, 14 **B** and 14 **M**, each having a diameter less than 3 cm were selected	Correctly identify all but three **B** nodules, but did not misclassify any **M** nodule

Kawata et al. [137]	To characterize the internal structure of small pulmonary nodules	Using multiscale curvature-based shape spectrum	Thin-section CT images of 27 pulmonary nodules (9 solid **B** and 18 solid and infiltrative **M** cases)	The distribution of the nodule characteristics in the feature space shows good evidence of separation between the two classes

McNitt-Gray et al. [231]	To classify nodules into benign or malignant	LDA with stepwise feature selection based on nodule's shape, size, attenuation, distribution of attenuation, and texture	HRCT scans of 17 **M** and 14 **B** nodules	Correct classification rate of 90.3%

Kawata et al. [232]	To discriminate between **B** and **M** nodules	LDA with stepwise feature selection based on nodule's features (density and curvatures) and surrounding structure features	CT images of 248 pulmonary nodules including 179 **M** and 69 **B** nodules	Nodule's features (*A*_*z*_ = 0.88) were more effective than the surrounding structure features (*A*_*z*_ = 0.69) in classification. Combing both features achieves *A*_*z*_ = 0.94

Kawata et al. [233]	To obtain nodule diagnosis information by image retrieval from a database of known diagnosis	Retrieving the nodules with similar characteristics from a 3D image database based on its CT density and curvature index	CT images of 248 pulmonary nodules including 179 **M** and 69 **B** nodules	The resulted visual figures are sorted from more similar to less similar with **M** case and show a high similarity with the test nodule

Matsuki et al. [234]	To classify nodules into benign or malignant	ANN with 16 subjective features determined by radiologists and 7 clinical data	155 HRCT scans of 99 **M** and 56 **B** nodules	*A*_*z*_ = 0.951 in a leave-one-out test

Lo et al. [235]	To quantify lung nodules in thoracic CT	A NNs based on geometrical features, intensity, and texture features	CT images of 48 cases of lung nodules (24 **B**, 24 **M**)	*A*_*z*_ = 0.89

Aoyama et al. [236]	To classify nodules into benign or malignant	LDA with Wilks' lambda stepwise feature selection	Thick-slice (10 mm) screening LDCT scans of 76 **M** and 413 **B** nodules	*A*_*z*_ = 0.846 in a leave-one-out test

Nakamura al. [237]	To classify nodules into benign or malignant	Two NNs: one trained with 8 subjective features recorded by radiologist rating and the other with 12 matched computerized objective features	56 radiographs of 34 **M** and 22 **B** nodules	*A*_*z*_ = 0.854 using subjective features and *A*_*z*_ = 0.761 using objective features. The reported radiologist accuracy was *A*_*z*_ = 0.752

Iwano et al. [238]	To classify the shape of pulmonary nodules using computer analysis of HRCT	LDA with 2 features (circularity and second moment)	HRCT images from 102 patients with 102 nodules classified as round or oval, lobulated, polygonal, tentacular, speculated, ragged, and irregular	For 95 of 102 cases, the shape classification by the two radiologists was the same. For the seven mismatched cases, pulmonary nodules with circularity ≤0.75 and second moment ≤0.18 were very likely to reveal lung cancer

Shah et al. [239]	To classify nodules into benign or malignant	Logistic regression or QDA with stepwise feature selection from 31 features	Thin-slice (≤3 mm) CE-CT scans of 19 **M** and 16 **B** nodules	*A*_*z*_ = 0.69 and 0.92 with logistic regression and QDA, respectively, in a leave-one-out test

Matsuoka et al. [240]	To analyze features of peripheral noncalcified solitary pulmonary nodules in patients with emphysema	Analyze the fractal dimensions of the nodule interfaces, nodule circularity, and the percentage of the nodule surrounded by emphysema	CT images of 41 nodules (21 **M**, 20 **B**) in 41 patients with emphysema	In patients with emphysema, there were no significant differences in fractal dimension, circularity, or frequency of lobulation or spiculation between **M** and **B** nodules

Mori et al. [241]	To classify nodules into benign or malignant	LDA using 3 features: shape index, curvedness values, and attenuation	Thin-slice (2 mm) CE-CT scans of 35 **M** and 27 **B** nodules	*A*_*z*_ = 0.91 and 1.0 with non-CE CT and CE-CT, respectively, in a leave-one-out test

Suzuki et al. [117]	To classify nodules into Benign or Malignant	Multiple MTANNs using pixel values in a 9 × 9 subregion	Thick-slice (10 mm) screening LDCT scans of 76 **M** and 413 **B** nodules	*A*_*z*_ = 0.88 in a leave-one-out test

Iwano et al. [242]	To classify nodules into benign or malignant	LDA based on nodule's circularity and second moment features	HRCT (0.5–1 mm slice) scans of 52 **M** and 55 **B** nodules	Sensitivity of 76.9% and a specificity of 80%

Way et al. [243]	To classify nodules into benign or malignant	LDA or SVM with stepwise feature selection	CT scans of 124 **M** and 132 **B** nodules in 152 patients	*A*_*z*_ = 0.857 in a leave-one-out test

Chen et al. [244]	To classify nodules into benign or malignant	ANN ensemble	CT scans (slice thickness of 2.5 or 5 mm) of 19 **M** and 13 **B** nodules	*A*_*z*_ = 0.915 in a leave-one-out test

Lee et al. [245]	To classify nodules into benign or malignant	GA-based feature selection and a random subspace method	Thick-slice (5 mm) CT scans of 62 **M** and 63 **B** nodules	*A*_*z*_ = 0.889 in a leave-one-out test

El-Baz et al. [246]	To classify nodules into benign or malignant	Analysis of the spatial distribution of the nodule Hounsfield values	CT scans (2 mm slice) of 51 **M** and 58 **B** nodules	Sensitivity of 92.3% and a specificity of 96.6%

El-Baz et al. [247]	To classify nodules into benign or malignant	Analysis of the SHs needed to delineate the lung nodule	CT scans (2 mm slice) of 153 **M**and 174 **B** nodules	*A*_*z*_ = 0.9782

**Table 10:** Evaluation of nodule malignancy in PET.

Study	Database	Accu./PPV	Sensitivity	Specificity
Dewan et al. [319]	30	PPV = 90%	95%	80%
Gupta et al. [320]	61	PPV = 92%	93%	88%
Lowe et al. [321]	89	Accu. = 91%	92%	90%
Lee et al. [322]	71	PPV = 86%	95%	82%
Herder et al. [323]	36	PPV = 72%	93%	77%
Halley et al. [324]	28	NA	94%	89%

*Accu denotes accuracy and PPV denotes positive productive value.

**Table 11:** Evaluation of nodule malignancy in fused PET/CT.

Study	Database	Accu./PPV	Sensitivity	Specificity
Keidar et al. [325]	42	PPV = 89%	96%	82%
Yi et al. [326]	119	Accu. = 93%	96%	88%
Nie et al. [327]	92	Accu. = 95%	NA	NA
Nakamoto et al. [328]	53	Accu. = 87%	94%	75%

*Accu. denotes accuracy and PPV denotes positive productive value.

## Abbreviations

1D: One dimensional
2D: Two dimensional
3D: Three dimensional
4D: Four dimensional
A: Automatic
AAH: Atypical adenomatous hyperplasia
AAM: Active appearance model
AC: Active contour
Acc.: Accuracy
AD: Absolute surface distance
ADT: Adaptive doubling time
AL: Automation level
ANN: Artificial neural network
ASM: Active shape model
*A*_*z*_: Area under the ROC curve
B: Benign
BAC: Bronchioloalveolar carcinoma
CAD, CADx: Computer-aided diagnosis
CADe: Computer-aided detection
CART: Classification and regression tree
CE-CT: Contrast-enhanced CT
CCL: Connected component labeling
CT: Computed tomography
DC: Discriminative classification
Dim: Dimension
DM: Deformable model
DP: Dynamic programming
DSC: Dice similarity coefficient
ELCAP: Early Lung Cancer Action Program
EM: Expectation-maximization
FCM: Fuzzy C-means
FDG: Fluorodeoxyglucose (^18^F)
FHMC: Fuzzy hidden markov chain
FLAB: Fuzzy locally adaptive bayesian
FN: False negative
FP: False positive
FPNs: False positive nodules
GAD: Growth analysis from density
GC: Graph-cut
GGO: Ground-glass opacity
GLCM: Grey-level cooccurrence matrix
GMMs: Gaussian mixture models
GR: Growth rate
GS: Gold standard
GT: Ground truth
GTV: Gross tumor volume
HD: Hausdorff distance
HR: High-resolution
HRCT: High-resolution CT
HU: Hounsfield
ICM: Iterative conditional mode
KDE: Kernel density estimator
KNN: K-nearest neighbor
LAP-MTANN: Laplacian eigenfunction ANN
LBP: Local binary pattern
LCDG: Linear combination of discrete Gaussian
LDA: Linear discriminant analysis
LDC: Linear discriminant classifier
LDCT: Low-dose computed tomography
LIDC: Lung Image Database Consortium
LoG: Laplacian of Gaussian
LRT: Likelihood Ratio Test
LS: Level set
M: Malignant
MDCC: Maximum distance inside connected components
MTANNs: Massive-training ANNs
MTSVR: Massive-training support vector regression
MAP: Maximum A Posteriori
MGRF: Markov Gibbs random field
ML: Maximum likelihood
MLN: Mediastinallymph node
MM: Mathematical morphology
MO: Morphological operations
MRF: Markov random field
MS: Mean shift
NNs: Neural networks
NA: Nonapplicable
NSCLC: Non-small-cell lung cancer
OM: Overlap measure
PA: Posterior-anterior
PC: Probabilistic classification
PCA: Principle component analysis
PET: Positron emission tomography
PML: Pixel machine learning
PPV: Positive predictive value
PSR: Pleural surface removal
PVE: Partial-volume effect
PVM: Partial volume method
QDA: Quadratic discriminant analysis
RAGF: Robust anisotropic Gaussian fitting
RECIST: Response evaluation criteria in solid tumors
RG: Region growing
RmsD: Root mean square distance
ROC: Receiver-operating-characteristics
ROI: Region of interest
MODALS: Multiple-object detection and location system
SCD: Symmetrical point-to-mesh comparison distance
Sen.: Sensitivity
SH: Spherical harmonic
SMDC: Selective marking and depth-constrained
SNO: Surface normal overlap
SPD: Signed border positioning distance
Spec.: Specificity
SPN: Solitary pulmonary nodule
SPVA: Segmentation-based partial volume analysis
SUV: Standard uptake value
SVM: Supported vector machine
TH: Thresholding
TN: True negative
TP: True positive
TPNs: True-positive nodules
UI: User interactive
VOI: Volume of interest
WHO: World Health Organization
WS: Watershed.
